# New Insights on Eggplant/Tomato/Pepper Synteny and Identification of Eggplant and Pepper Orthologous QTL

**DOI:** 10.3389/fpls.2016.01031

**Published:** 2016-07-18

**Authors:** Riccardo Rinaldi, Allen Van Deynze, Ezio Portis, Giuseppe L. Rotino, Laura Toppino, Theresa Hill, Hamid Ashrafi, Lorenzo Barchi, Sergio Lanteri

**Affiliations:** ^1^DISAFA Plant Genetics and Breeding, University of TurinTurin, Italy; ^2^Seed Biotechnology Center, University of California, DavisDavis, CA, USA; ^3^CREA-ORL Research Unit for Vegetable CropsMontanaso Lombardo, Italy

**Keywords:** orthology, chromosome rearrangement, CDS, gene alignment, QTL, genetic map

## Abstract

Eggplant, pepper, and tomato are the most exploited berry-producing vegetables within the Solanaceae family. Their genomes differ in size, but each has 12 chromosomes which have undergone rearrangements causing a redistribution of loci. The genome sequences of all three species are available but differ in coverage, assembly quality and percentage of anchorage. Determining their syntenic relationship and QTL orthology will contribute to exploit genomic resources and genetic data for key agronomic traits. The syntenic analysis between tomato and pepper based on the alignment of 34,727 tomato CDS to the pepper genome sequence, identified 19,734 unique hits. The resulting synteny map confirmed the 14 inversions and 10 translocations previously documented, but also highlighted 3 new translocations and 4 major new inversions. Furthermore, each of the 12 chromosomes exhibited a number of rearrangements involving small regions of 0.5–0.7 Mbp. Due to high fragmentation of the publicly available eggplant genome sequence, physical localization of most eggplant QTL was not possible, thus, we compared the organization of the eggplant genetic map with the genome sequence of both tomato and pepper. The eggplant/tomato syntenic map confirmed all the 10 translocations but only 9 of the 14 known inversions; on the other hand, a newly detected inversion was recognized while another one was not confirmed. The eggplant/pepper syntenic map confirmed 10 translocations and 8 inversions already detected and suggested a putative new translocation. In order to perform the assessment of eggplant and pepper QTL orthology, the eggplant and pepper sequence-based markers located in their respective genetic map were aligned onto the pepper genome. GBrowse in pepper was used as reference platform for QTL positioning. A set of 151 pepper QTL were located as well as 212 eggplant QTL, including 76 major QTL (PVE ≥ 10%) affecting key agronomic traits. Most were confirmed to cluster in orthologous chromosomal regions. Our results highlight that the availability of genome sequences for an increasing number of crop species and the development of “ultra-dense” physical maps provide new and key tools for detailed syntenic and orthology studies between related plant species.

## Introduction

Eggplant (*Solanum melongena* L.) pepper (*Capsicum annuum* L.) and tomato (*Solanum lycopersicum* L.) are the most exploited berry-producing vegetables within the Solanaceae family, which comprises over 3000 species. The genomes of the three species differ in size, but share a similar gene number (~35,000). Moreover, each has 12 chromosomes which have undergone inversions as well as inter- and intra-chromosomal translocations causing a redistribution of loci. The three species provide hence a model for exploring the basis of phenotypic diversity and adaptation to agricultural environments.

The whole-genome sequence of tomato (The Tomato genome Consortium, 2012) was the first to be published, followed by two chili pepper genomes (Kim et al., 2014; Qin et al., 2014). More recently, an eggplant draft genome has also been released (Hirakawa et al., 2014).

Synteny has been studied quite extensively within the Solanaceae family. Closely related species, such as tomato and potato, have been found to have a highly conserved marker order that is modified by clearly-defined events such as paracentric inversions (Bonierbale et al., 1988; Tanksley et al., 1992). Wu et al. (2009a) conducted a comparison between eggplant and tomato maps based on 289 orthologous markers, and identified at least 24 inversions (two per chromosome on average), and 5 translocations differentiating the two species. At greater evolutionary distances, as in tomato and pepper, the chromosome number remains unchanged but inter- and intra-chromosomal translocations and inversions have redistributed and repositioned loci, and shorter syntenic blocks were found (Tanksley et al., 1988; Wu et al., 2009b). In particular, Wu et al. (2009b) identified 299 syntenic markers and detected at least 19 inversions and 6 chromosome translocations that differentiating the two species.

Overall, as highlighted by Wu and Tanksley (2010), members of the Solanaceae family have undergone a modest rate of chromosomal change and non-random positioning of the chromosomal rearrangement breakpoints compared to other plant families.

In pepper, and to a lesser extent in eggplant, the inheritance of agronomic traits has been studied intensively, and a growing number of genes and QTL have been identified and sometimes the underlying genes isolated (Grandillo et al., 1996, 1999; Frary et al., 2000; Chaim et al., 2001, 2003; Rao et al., 2003; Zygier et al., 2005; Barchi et al., 2007; Bradshaw et al., 2008; Huang and van der Knaap, 2011; Zhang et al., 2012).

In pepper, several linkage maps have been developed, based on both intraspecific and interspecific populations, and genotyped with various marker systems. Some of them also permitted the location of QTL associated with key breeding traits (Tanksley et al., 1988; Livingstone et al., 1999; Kang et al., 2001; Paran et al., 2004; Sugita et al., 2005, 2013; Minamiyama et al., 2006; Yi et al., 2006; Barchi et al., 2007; Wu et al., 2009b; Lu et al., 2012; Mimura et al., 2012; Kim et al., 2014; Park et al., 2014; Qin et al., 2014). Recently Yarnes et al. (2013) identified QTL for capsaicinoids, fruit quality, and plant architecture-related traits in an interspecific RIL population from a cross between *Capsicum frutescens* and *C. annuum;* while Li et al. (2015), by resequencing two *C. annuum* lines, developed an indel-based linkage map which was anchored to the physical map of the Zunla-1 reference genome (Qin et al., 2014). Furthermore, two high quality EST-based *Capsicum* genetic maps have been produced using GeneChip technology—a 16 K unigene interspecific and a 5.6 K unigene intraspecific map (Hill et al., 2015).

In eggplant some QTL were located on a map based on an F_2_ interspecific population (Doganlar et al., 2002b; Frary et al., 2003), while intraspecific populations were the basis for mapping two QTL underpinning parthenocarpy (Miyatake et al., 2012), as well as a single dominant gene and a QTL conferring resistance to *Ralstonia solanacearum* (Lebeau et al., 2011). More recently, a densely-populated intraspecific linkage map based on RAD-tag-derived marker genotyping of an intraspecific F_2_ population has been developed, and used for identifying QTL affecting anthocyanin content and key agronomic traits (Barchi et al., 2011; Portis et al., 2014). Using a GWAS approach, the previously identified loci were validated and new marker/trait associations were detected (Cericola et al., 2014; Portis et al., 2015).

Based on available genetic maps, synteny among Solanaceae has been extensively studied during the last three decades. However, the recent progresses on both sequencing technologies and assembly algorithms have enabled the release of genome sequences in many species.

The goal of the present study was to infer on the syntenic relationships between eggplant, pepper and tomato based on the availability of their genome sequence, as well as to perform the first assessment of eggplant and pepper orthologous QTL influencing key breeding traits. The reported results will provide a backbone platform for future genomic selection programs within the Solanaceae family.

## Materials and methods

### Retrieving and alignment of genome data

We retrieved from publicly available databases: (i) the pepper genome sequence (CM334 v1.55), its annotation, and the CDS produced by the Plant Genomics and Breeding Institute (Seoul National University:); (ii) the tomato genomic assembly (ITAG2.5) and the CDS (ITAG2.3) provided by the International Tomato Genome Sequencing Consortium (ftp://ftp.solgenomics.net/tomato_genome); (iii) the eggplant draft genome assembly (SME_r2.5.1) supplied by the Kazusa DNA Research Institute (ftp://ftp.kazusa.or.jp/pub/eggplant/).

The CDS of pepper (34,899) and tomato (34,727) were aligned to the tomato ITAG2.5 and the pepper v1.55 genome sequence, respectively, using GMAP with default parameters (Wu and Watanabe, 2005). The resulting matches were filtered using a cut-off threshold of 80% identity and 75% coverage, with a minimum alignment length of 200 bp (Hill et al., 2015). Multiple hits per CDS were filtered out, retaining in single copy the hit with the highest match, identity and coverage. To identify multiple queries aligned on the same match, the hits lying on the same locus were detected with a custom Python script. The script was set with a confidence interval (CI) of 25 bp, thus partial alignments were also included. The filtered alignment of pepper CDS to the tomato genome and the tomato CDS to pepper genome were termed PeCDS/ToG and ToCDS/PeG, respectively. Translocations and inversions were considered as such when pepper genome regions spanning at least 0.5 Mb were involved.

The 347 COSII primers from Wu and Tanksley (2010) and Wu et al. (2009a,b) were aligned to pepper and tomato genomes using Blastn (Camacho et al., 2009) with default settings. The output was processed with the custom Python pipeline. For each COSII marker only the forward and reverse primers which aligned on the same chromosome and at a distance analogous to the COS length reported by Wu and Tanksley (2010) and Wu et al. (2009a,b) were retained and included in the synteny map.

The 475 pepper unigene markers previously used to develop the pepper genetic map and perform QTL analysis (later referred to as PeM) (Yarnes et al., 2013) were aligned on the pepper genome v1.55 (Kim et al., 2014) with GMAP default settings. The GFF3 output file was filtered for a minimum of 98% identity and 200 matching bp and not more than 50 bp of mismatching (Hill et al., 2015). The not-matching sequences were excluded from further analyses. The alignment of pepper markers to the pepper genome is referred to as PeM/PeG.

The 339 eggplant RAD-tag sequences together with further 40 microsatellites, 27 COSII primers, 6 RFLPs and 1 CAPS which were previously located in the eggplant genetic map (Barchi et al., 2012) were combined to obtain a final dataset of 413 sequences (EgM). By using GMAP with default settings, these were aligned to the available genomes of eggplant (EgM/EgG), tomato (EgM/ToG), and pepper (EgM/PeG) (The Tomato genome Consortium, 2012; Hirakawa et al., 2014; Kim et al., 2014). For each species, the GFF3 output files were processed through a custom Python pipeline and high confidence hits selected. The alignment EgM/EgG was filtered using a minimum of 75% identity and 100 matching bp and not more than 50 bp of mismatching as cut-off parameters, while the alignments EgM/ToG and EgM/PeG were filtered at 75% identity and 40% coverage, with a minimum alignment length of 200 bp. When multiple hits were obtained for a given marker, only the alignment with the highest number of matches was retained. To improve the number of aligned markers, unmatched sequences were aligned with Blastn (Camacho et al., 2009) and manually screened to filter out poor quality sequences and misalignments. With the goal of increasing the percentage of eggplant anchored genome in a newly developed map, the not previously anchored scaffolds by Hirakawa et al. (2014) but including markers mapped by Barchi et al. (2012), were also anchored and, when possible, correctly oriented.

### Development of a pepper GBrowse and assessment of synteny

The GMOD GBrowse viewer in combination with a MySQL database management system were used to store, search and display the gene annotations of pepper (Kim et al., 2014) and PeM/PeG EgM/PeG alignments.

The files were uploaded to a MySQL database using the Perl pipeline provided by the program. The GBrowse web page provided information on gene structure and functions, gene ontology, and position and sequence of molecular markers. The positions of the markers were visually screened by keyword searching and the most probable position of each marker on the developed genetic map was identified.

The alignments PeCDS/ToG and ToCDS/PeG as well as EgM/ToG and EgM/PeG were used for the development of four syntenic maps. In each of them the CDS or marker sequences of tomato, eggplant and pepper were positioned in respect to the reference physical or genetic map and the newly detected location following the alignment. **Figures 3–5** report the syntenic maps: EgM/ToG, EgM/PeG, and ToCDS/PeG, respectively, in which marker sequences or CDS positions are reported on Y axis while the newly detected aligned genome location on X axis.

For the ToCDS/PeG and EgM/PeG alignments the developed pepper GBrowse was used as a reference platform.

### Assessment of QTL orthology between eggplant and pepper

The markers associated with QTL previously identified in eggplant (Barchi et al., 2012; Portis et al., 2014, 2015) and pepper (Yarnes et al., 2013) (Supplementary Tables S1, S2) were used for further analyses based on their alignment.

The CI (confidence interval) of pepper and eggplant QTL were transformed into physical units (bp) according to position in the PeM/PeG and EgM/EgG alignments, respectively, and the distances recorded on the genetic map (Barchi et al., 2012; Yarnes et al., 2013).

QTL orthology between eggplant and pepper was assessed by locating the position, on the pepper genome, of eggplant (Barchi et al., 2012; Portis et al., 2014, 2015) and pepper markers (Yarnes et al., 2013) associated with the QTL (Supplementary Tables S1, S2). As detection of pepper QTL was based on phenotypic data collected in two environments, the average of the two CI (Yarnes et al., 2013) was taken for further analysis. The transformation of centiMorgans (cM) to base pairs (bp) was obtained via the ratio Δ_bp∕_Δ_cM_, where Δ_bp_ is the distance in base pairs between two neighboring markers while Δ_cM_ is their distance in cM. The physical CI were then obtained by multiplying the number of bp/cM for the LOD confidence interval of each QTL peak.

All aligned eggplant markers associated with an eggplant QTL were manually screened and, when the QTL from each species influenced related traits, the one lying within or in CI proximity to a pepper QTL was retained. QTL clusters associated to related traits were retained. The final comparative map was drawn using MapChart v2.1 (Voorrips, 2002) according to the marker position obtained from the alignment.

## Results and discussion

### Tomato and pepper synteny

Although the alignment of intra- and inter-specific pepper maps with the tomato genome has recently been performed (Hill et al., 2015), we report on the first alignment between the CDS and genomes of the two species.

Two high quality genome sequences have been recently released (Kim et al., 2014; Qin et al., 2014). We based our study on the genome sequence of Kim et al. (2014), since its anchoring was performed on the bases the high-density pepper genetic map we used in our study (Yarnes et al., 2013).

**Figure 1:**
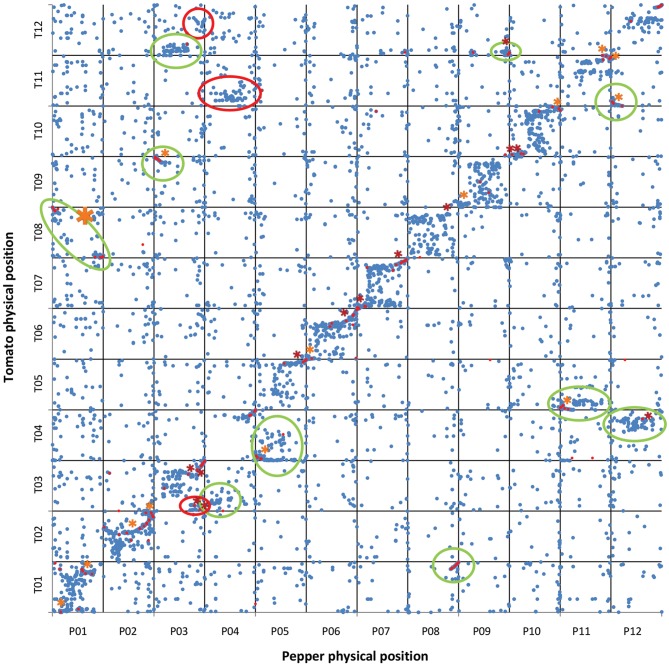
**Tomato CDS vs. pepper genome sequence**. Physical positions of tomato CDS (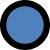) matching pepper genome sequences with ≥80% ID and ≥75% coverage. Physical positions of COSII markers (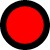) matching tomato and pepper genome sequences. The physical location of the sequences in tomato are shown on the vertical axis, with the pepper genome on the horizontal axis. A total of 19,734 CDS were mapped on pepper chromosomes. The translocations and inversions previously reported in literature are circled in green and marked with an orange asterisk, respectively. The newly identified translocations and inversions are circled in red and marked with a red asterisk, respectively.

The 34,727 tomato CDS aligned against the pepper genome v1.55 sequence resulted in 51,448 matches. The filtering retained 23,735 hits of which 19,734 were unique and were included in ToCDS/PeG. Likewise, a total of 34,899 pepper CDS were aligned against tomato ITAG2.5 genome assembly resulting in 52,737 matches. After filtering 27,417 were retained, of these 20,700 were unique and were included in PeCDS/ToG. The filtering removed multiple matches of the same query and a custom Python pipeline was programmed to screen alignments of multiple queries to the same matching sequence. The scripts screened the ToCDS/PeG and PeCDS/ToG coordinates in order to detect multiple genes aligned to the same match or its proximity (within 50 bp). In ToCDS/PeG a total of 2145 genes giving rise to non-unique matches were identified, while in PeCDS/ToG there were 3679. These discrepancies might be due to paralog genes aligned to the same orthologous gene as well as to the alignment of several CDS to non-coding sequences. In order to better assess rearrangements between the two species, it was essential to minimize the number of false matches, but since it was impossible to distinguish the false queries of each group of matches, their removal was impossible. Thus, the lower redundancy of ToCDS/PeG led us to choose it as the reference for further analyses. The dot plots showing relative tomato vs. pepper physical positions on each of the 12 chromosomes are reported in Figure 1. Of the 19,734 aligned sequences, 11,919 (60.4%) were aligned on the same chromosome.

Wu and Tanksley (2010) and Wu et al. (2009a,b) previously developed a synteny map of eggplant, pepper and tomato based on COSII markers. Using Blastn (Camacho et al., 2009) we aligned the 347 forward and reverse COSII primers on pepper and tomato chromosomes and (presumably due to their shortness) 30,942 matches were obtained. Processing with a custom Python pipeline was employed to locate their most likely position on each genome, and only those lying on the same chromosome were retained. Then, the distance between each pair of forward and reverse primers was compared with the length of the corresponding COSII marker developed by Wu and Tanksley (2010) and Wu et al. (2009a,b) and 160 were retained and included in the map (Figure 1).

Our results complement those of Hill et al. (2015), which provided insights on the genetic position of rearrangements between pepper and tomato. However, thanks to availability of both CDS and genome sequences of pepper and tomato, we were able to develop a more detailed synteny map as well as to recognize previously-unidentified small rearrangements.

Our results confirm 10 translocations and 14 inversions previously reported (Livingstone et al., 1999; Wu et al., 2009b; Wu and Tanksley, 2010; Qin et al., 2014). Additionally, 3 new translocations and 13 new inversions were detected. However, our results did not confirm 4 previously reported inversions (2 on P01, one on the translocation between T03/P04 and one on P11 (Wu et al., 2009b; Wu and Tanksley, 2010), while a new translocation on lower P03 was identified (Figure 1). In addition, we did not observe several small translocations previously reported in centromere regions (e.g., the translocation between T02, T08, and P02, and the translocation between T03 and upper P06 (Wu et al., 2009b). This might be due to errors in the genomic mapping of these regions and/or to software misalignment in repetitive regions. In agreement with Hill et al. (2015), we highlighted orthology between the P04 centromere and T11 (Figure 1) which, together with the already reported translocation between upper P04 and T03 (Hill et al., 2015), confirms that there have been at least 2 translocation events within the non-recombining region of P04. P03 was found to be consist of upper T09 plus an unreported non-recombining region shared between T03/T12 and lower T03, while a duplication was confirmed to have occurred between lower P03 and T12 (Figure 1). Unlike Hill et al. (2015) we did not detect a translocation between P04 and T12 (Figure 1).

**Figure 2:**
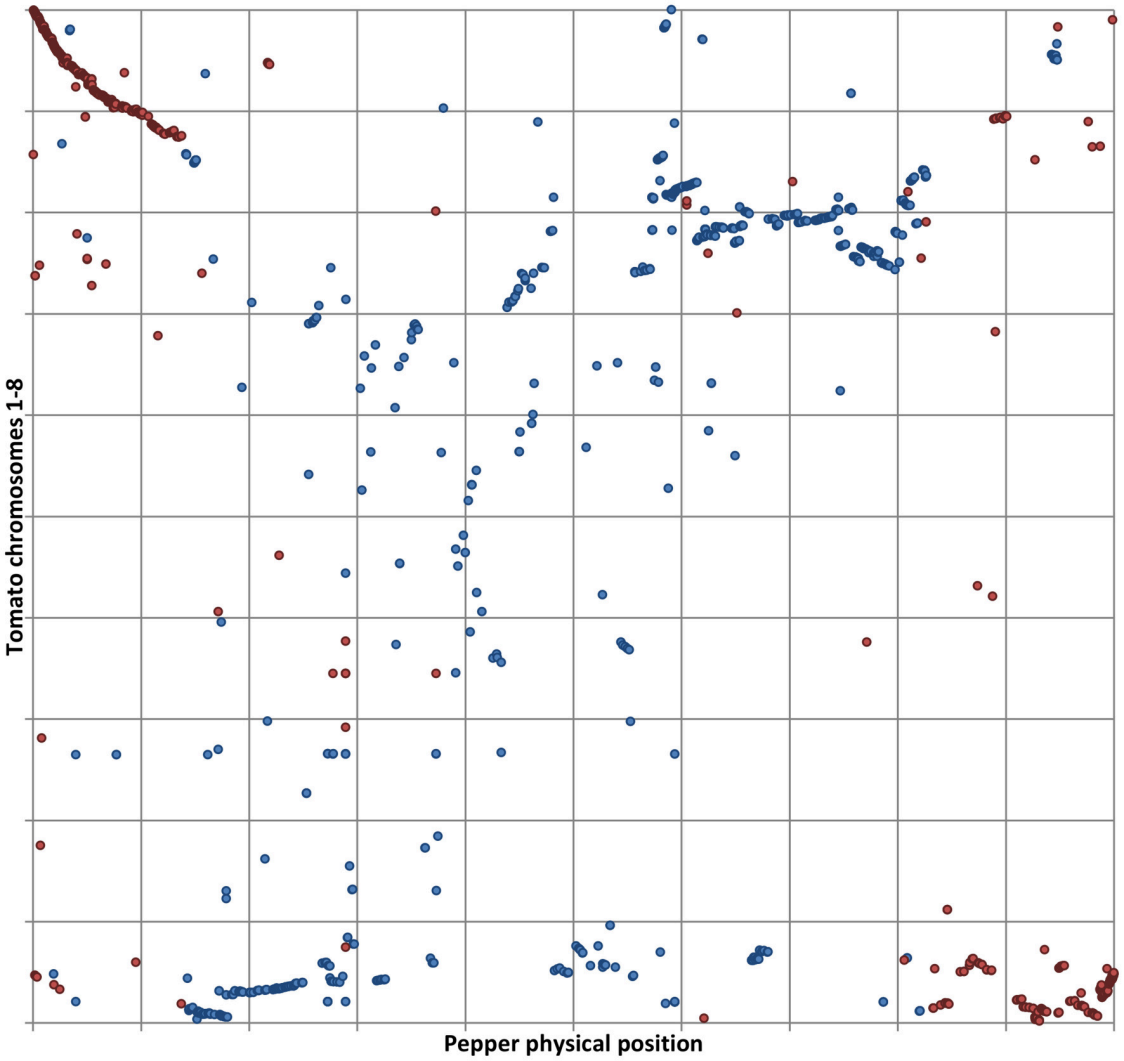
**Details of tomato CDS aligned to P01**. Physical positions of tomato CDS matching pepper genome with ≥80% ID and ≥75% coverage. The relative position of tomato CDS belonging to T01 (in blue) and T08 (in red) is shown on the vertical axis. The horizontal axis shows the normalized P01. Matches belonging to T01 are in blue, matches from T08 in red.

Our results also reinforce the previously proposed hypothesis that an illegitimate pairing and crossing over event occurred in relatively recent times between two non-homologous, metacentric chromosomes in the ancestral genome of *C. annuum* (Tanksley, 1984; Tanksley et al., 1988; Livingstone et al., 1999; Wu et al., 2009b; Wu and Tanksley, 2010; Qin et al., 2014), as we also detected the translocation involving the distal arms of P01 and T08 (Figures 1, 2). As already noted, the outcome of the reciprocal exchange corresponds to P01 (submetacentric) and P08 (acrocentric) in the genome of cultivated *C. annuum* (Tanksley, 1984; Tanksley et al., 1988; Livingstone et al., 1999; Wu et al., 2009b; Wu and Tanksley, 2010; Qin et al., 2014); however we were also able to identify the putative position of the rearrangement, around 37–38 and 225–227 Mbp of P01, as reported in Figure 1 and detailed in Figure 2.

The high definition of our ToCDS/PeG map highlighted a large number of small inversions, translocations, and transpositions mainly located in centromeric regions (e.g., P03 between T12 and T03; P05 between T04 and T05; P05 between T05 and T11; P12 between T12 and T04—see Supplementary Figure S1). It cannot be excluded that some of these small rearrangements might be the result of errors in scaffold anchoring and orientation due to the many repetitive sequences in plant and animal centromeres, which play a functional role in promoting concerted evolution of centromere DNA across chromosomes (Melters et al., 2013). However, repeated exchanges of genetic material between chromosomes, and transposon activity in untranslated regions, might be the causes of most of the small rearrangements we observed. This is particularly the case with pepper, in which the expansion of repetitive sequences in both heterochromatic and euchromatic regions is responsible for its genome size, which is approximately four-fold larger than in tomato (Kim et al., 2014).

Distances in genetic maps are based on recombination frequency and in regions characterized by low crossing over frequency, such as centromeres and telomeres, this may cause errors in the assessment of marker order and distances.

Our results highlight the improvement in analysis of their synteny allowed by the availability of a high quality genome sequence of both pepper and tomato. We were able not only to confirm the literature reports of chromosome rearrangements, but also to identify previously undetected translocations and inversions.

### Comparative analysis of eggplant genetic map and genome sequence

The eggplant markers (EgM) were aligned to the available eggplant draft genome (Hirakawa et al., 2014) in order to determine the physical position of genetic markers previously located in an intraspecific genetic map (Barchi et al., 2012) and to infer candidate genes lying within their CI. Most were RAD-tag markers, generated from DNA sequences flanking restriction sites throughout the genome (Barchi et al., 2011). Nevertheless, the sequences belonged to the same species, due to the lower conservation of interspersed genomic regions of these markers in respect to coding sequences, a relatively low value for identity and coverage were adopted.

Of the 413 eggplant markers previously mapped, 342 were aligned on the eggplant genome sequence (Hirakawa et al., 2014). Filtering retained 316 unique matches (77%), of which 103 were associated with QTL (Barchi et al., 2012; Portis et al., 2014, 2015). The positions of aligned markers and the corresponding scaffolds were ordered according to the eggplant map, but our attempts to determine the physical position of QTL we previously identified (Barchi et al., 2012; Portis et al., 2014, 2015) were not successful. A total of 276 scaffolds, corresponding to 28 Mbp [2.48% of the estimated total eggplant genome size of 1127 Mbp (Barchi et al., 2011; Delledonne et al., 2014; Hirakawa et al., 2014)] were ordered according to the genetic map. The average length of the sequences was 102,338 bp with an N50 of 146,573 bp. Gene prediction indicated a total of 3158 genes on the ordered scaffolds, of which 623 were transposable elements, pseudo, or short genes, or both. For the remaining 2535 genes (Table 1), the identification of putative candidate genes was not possible and transformation of their CI from genetic to physical units was obtained for just 14 markers, which were positioned in pairs on 7 scaffolds.

**Table 1:** **Statistics of the eggplant sequenced scaffolds bearing eggplant genetic markers**.

**Ordered SME_r2.5.1**	
Total sequence number	276
Total length (bp)	28,245,253 bp
Average length (bp)	102,338 bp
N50	146,573 bp
N90	57,379 bp
GC%	33.29%
Number of genes	2535
Number of transposable elements or pseudo genes	623

Fragmentation of the genome (N50 = 64,536 bp) and limited coverage (833 of 1124 Mbp) restricted the number of mapped markers we were able to align to the eggplant sequence and in most cases scaffolds were shorter than the CI of the mapped QTL.

Syntenic analyses and identification of QTL orthology between eggplant and pepper were thus based on our previously developed genetic map (Barchi et al., 2012).

### Collinearity of the eggplant map with the pepper and tomato genomes

**Figure 3:**
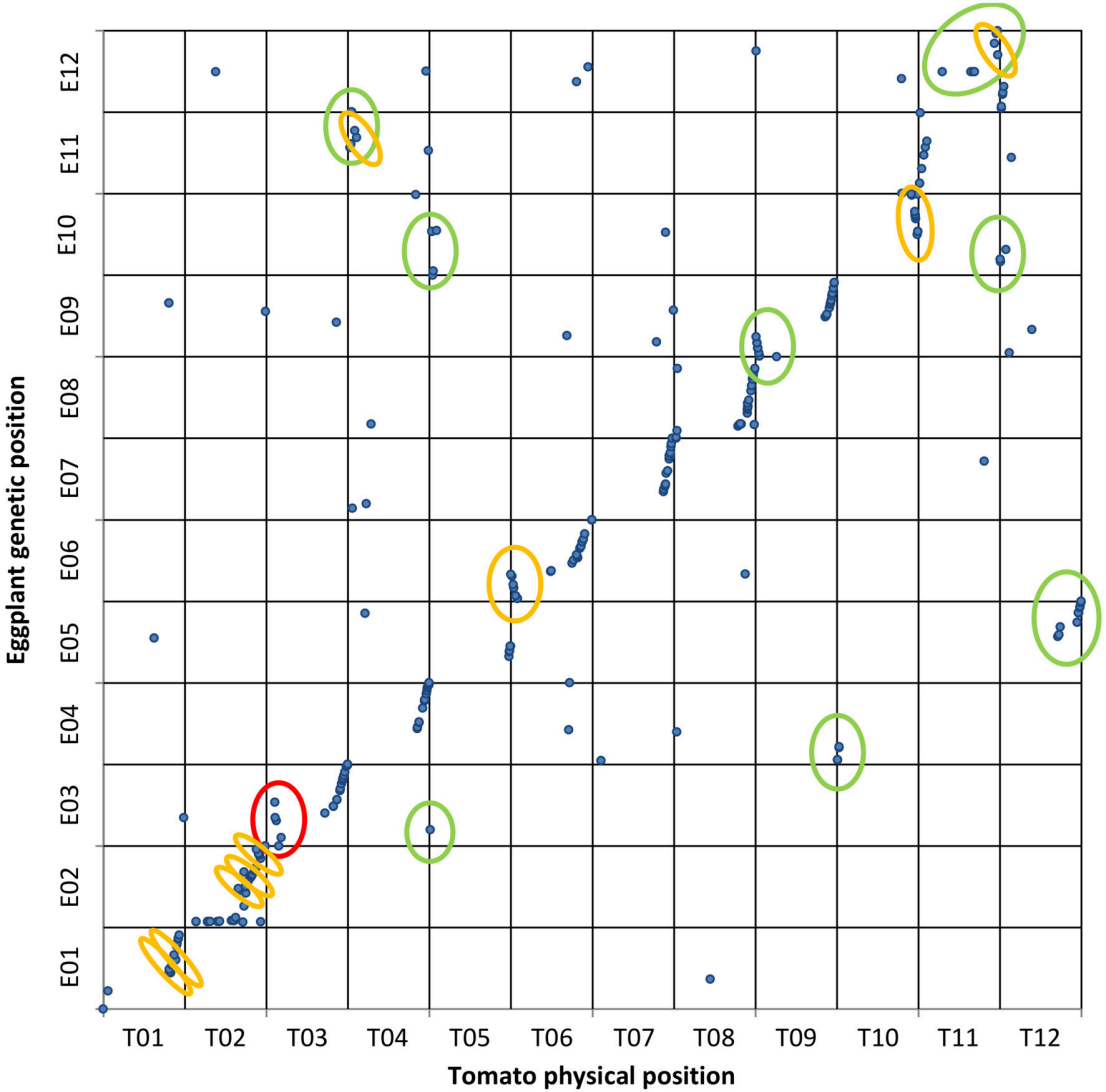
**Eggplant map vs. tomato genome**. Genetic and physical positions of eggplant RAD-tag markers (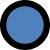) matching tomato genome sequences with ≥75% ID and ≥40% coverage. A set of 327 contigs were mapped on tomato chromosomes. The vertical axis shows the eggplant genetic map while the tomato genome is on the horizontal axis. The 7 translocations and 9 inversions previously reported are circled in green and orange, respectively. New rearrangements observed here are circled in red.

**Figure 4:**
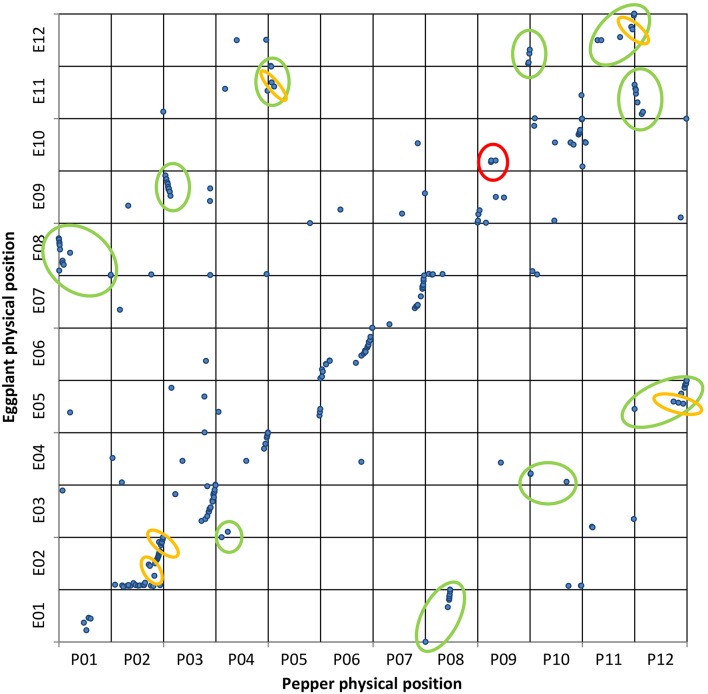
**Eggplant map vs. pepper genome**. Genetic and physical positions of eggplant (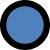) markers matching the pepper genome sequence with ≥75% ID and ≥40% coverage. A total of 313 markers were mapped onto pepper chromosomes. The vertical axis shows the eggplant genetic map, with the pepper genome on the horizontal axis. The translocations and inversions previously reported are circled in green and orange, respectively. The new putative translocation observed is circled in red.

Of the 413 eggplant marker sequences (Barchi et al., 2012) aligned to the ITAG2.50 tomato (The Tomato genome Consortium, 2012) and to the CM334 v1.55 pepper genome assemblies (Kim et al., 2014), 327 were positioned on tomato (79%) and 313 (76%) on pepper. We plotted the eggplant marker genetic positions vs. their physical positions on tomato (Figure 3) and pepper (Figure 4) genomes.

In the EgM/ToG dotplot (Figure 3) all 10 translocations previously identified on the basis of COSII markers in common between the two species (Wu et al., 2009b) were confirmed. The translocations between E03/T05 and E04/T10 included only one and two markers, respectively, presumably due to the low conservation of our randomly distributed eggplant genetic markers, which hampered the alignment of the majority of sequences. For the same reason only 9 of the 14 inversions previously identified by Wu et al. (2009b) were confirmed. Most of the others could not be assessed but, interestingly, we confirmed two translocated segments involving the lower arm of E11 and T04 as well as E12 and T11, with the latter having also an inverted small portion (Wu et al., 2009b). In addition, an unreported inversion was detected on upper chromosome 3.

The genetic positions of the eggplant markers plotted against their physical positions on the pepper genome (EgM/PeG) are reported in Figure 4. Our results, which to our knowledge represent the first direct syntenic analysis of the two species, were compared with syntenic analyses previously carried out among Solanaceae species (Wu and Tanksley, 2010), as well as between tomato and pepper (Wu et al., 2009b) and eggplant and tomato (Wu et al., 2009a).

We detected 14 translocated chromosomal segments; those involving E08/P01, E09/P03, and E11/P12 were also inverted. As expected only a portion of the translocated chromosomal segments involving E05/P12, E11/P05, and E12/P11 were also inverted, although our conclusions are based on alignments involving only two or three markers. Furthermore, two inversions involving chromosome 2 of both eggplant and pepper were also highlighted. Our results confirm what was expected on the basis of previous syntenic studies (Wu et al., 2009b), including the small inversion between P05 and E11, and therefore enables us to exclude the possibility that this result is due to misalignment. Interestingly, a translocation not detected in previous studies was also observed, involving three closely linked markers of the upper arms of chromosomes P09 and E10.

### QTL orthology between eggplant and pepper

The alignments against the pepper genome (Kim et al., 2014) of the 475 markers from the previously developed pepper genetic map (Yarnes et al., 2013) were filtered using relatively demanding requirements, since these markers belong to the same genetic map on which the scaffolds of the pepper genome sequence were anchored (Kim et al., 2014). The filtering retained 357 markers (75%). Of the total 175 markers influencing plant architectural, phenological, or fruit quality traits (Yarnes et al., 2013), we aligned 139 markers (79%). The position and CI of each QTL associated with the aligned markers were converted into physical units (bp).

**Figure 5:**
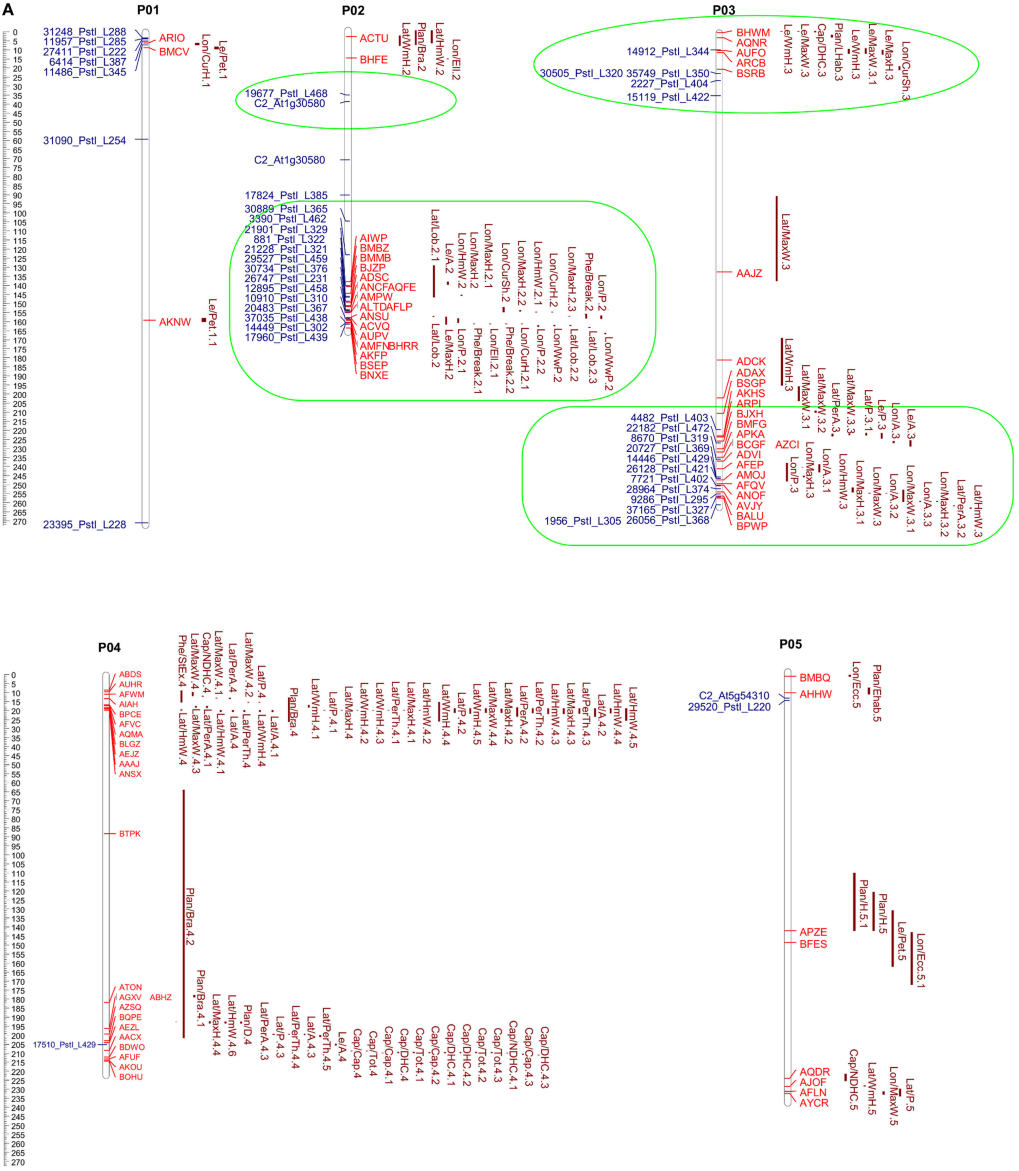

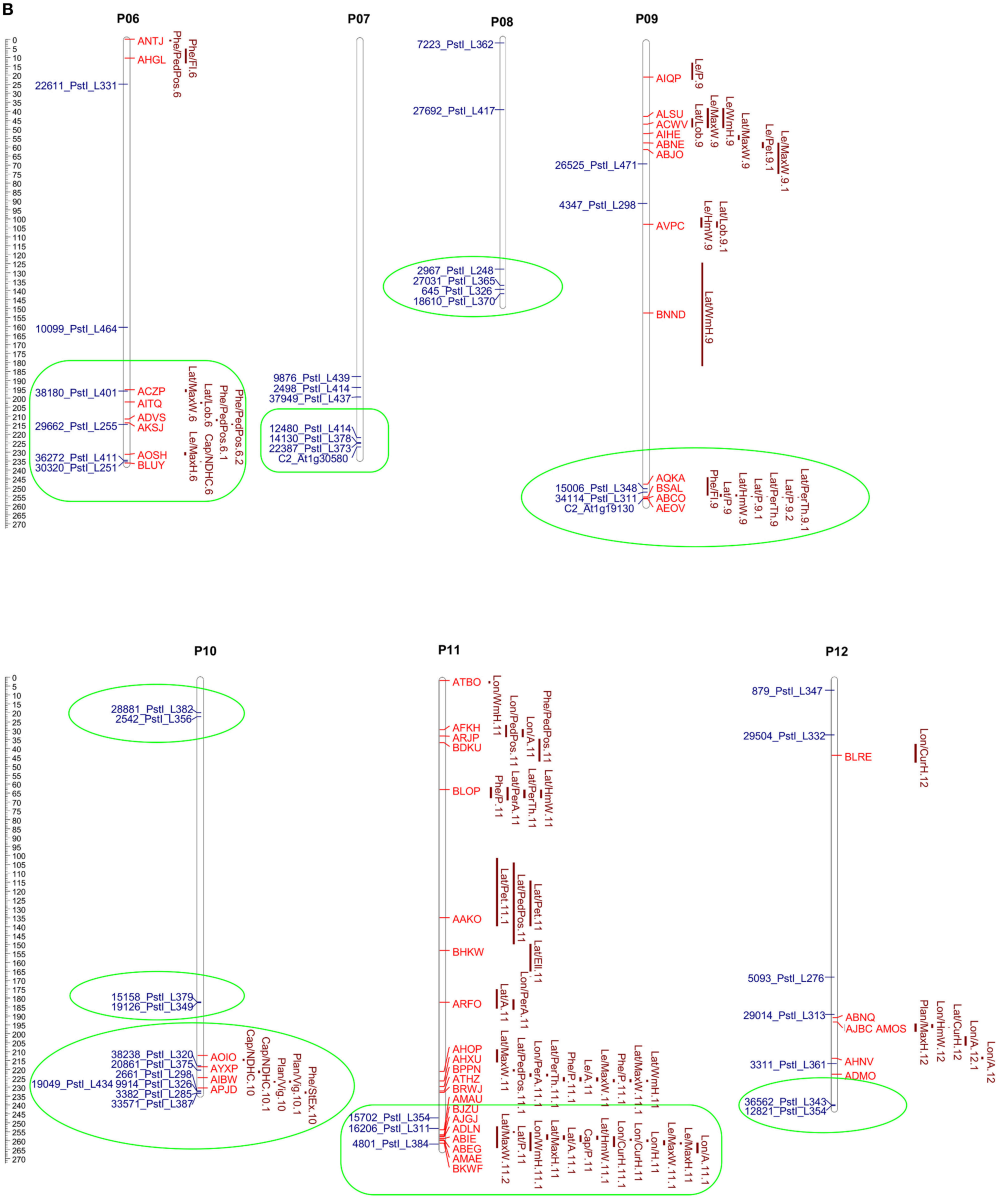
**(A,B)** Pepper QTL and eggplant marker locations. The scale shown on the left indicates the chromosome length in Mbp. Map positions of pepper QTL (Yarnes et al., 2013) are given on the right of each chromosome by the red bars. The length of the bars represents the QTL confidence interval. Eggplant marker names (Barchi et al., 2012) are shown to the left. The groups formed by QTL clustering are circled in green.

The 313 markers positioned in the eggplant genetic map developed by Barchi et al. (2012) and previously aligned with the pepper genome (Kim et al., 2014) (EgM/PeG) were screened in order to retain the eggplant markers aligning with pepper loci of potential interest. The specific “Pepper GBrowse” developed as tool for this research provided a valuable help in the visualization of the putative orthologous loci. A physical eggplant-pepper syntenic map was developed based on physical CI of pepper markers and the aligned orthologous eggplant markers (Figures 5A,B). This map locates a total of 88 eggplant markers associated with 212 eggplant QTL (Supplementary Table S3) and 114 pepper markers associated with 151 pepper QTL (Supplementary Table S4).

In order to detect orthologous QTL and find new regions of potential interest, the coordinates of all the eggplant markers, sorted by trait, are reported in Supplementary Table S3. Only major QTL (PVE ≥ 10) or QTL clusters potentially indicating new orthologous regions were considered. The regions in which eggplant and pepper markers formed clusters of QTL are reported in detail in Supplementary Figure S2, together with pepper QTL physical CI and the LOD peaks.

We identified 14 clusters of eggplant markers related to anthocyanin content, fruit, leaf, flower and plant phenology, and morphology. Eight of them are related to one or more orthologous QTL in pepper: i.e., P01 (interval 3–7 Mbp); P02 (127–169 Mbp); and P03 (216–256 Mbp) included eggplant and pepper QTL influencing peduncle, fruit and flowering time; P06 (195–235 Mbp), P07 (220–225 Mbp), P09 (245–247 Mbp), and P11 (243–258 Mbp) included QTL related to fruit shape and size; and P12 (~234 Mbp) contains traits related to the fruit peduncle (Supplementary Tables S5, S6, Figures 5A,B). The remaining 6 clusters: P02 (interval 36–40 Mbp); P03 (10–35 Mbp); P08 (134–136 Mbp); P10 (19–22 Mbp); P10 (~182 Mbp); and P10 (230–233 Mbp) (Supplementary Tables S5, S6, Figures 5A,B), co-localized multiple eggplant markers associated to an analogous QTL, though they have no counterpart in pepper. QTL clustering has previously been reported and might be due to inter-trait correlations or pleiotropy (Doganlar et al., 2002a; Portis et al., 2014, 2015).

As expected, the QTL affecting capsaicinoid content in pepper as well as the QTL affecting prickliness and anthocyanin pigmentation in eggplant did not find reciprocal counterparts.

As previously highlighted by micro-synteny analyses, the capsaicinoid-related genes in pepper emerged only after the final round of genome duplication and following their neo-functionalization (Kim et al., 2014). On the other hand, prickliness is a specific trait characterizing some eggplant genotypes, which underwent negative selection pressure during domestication with the goals both of avoiding damage to the fruit skin during plant growth and of facilitating harvest and post-harvest operations.

Our results show that QTL associated to prickliness of stem, calix, and leaf in eggplant clustered on upper P01 (3–7 Mbp), P02 (144–159 Mbp), P06 (195–235 Mbp), and P07 (195–220 Mbp), and co-localized with others related to the length of the fruit and of the peduncle in both pepper and eggplant (P01 3–7 Mbp; P02 144–159 Mbp; P06 195–235 Mbp, P07 186–220 Mbp). Although the reason is still unknown, we hypothesize that the clustering on the same genetic region of QTL controlling breeding traits may be related to the co-localization of genes involved in cell proliferation, cell elongation or both. Indeed the differential activity of these genes in the two species might promote the growth of the prickle in eggplant and the elongation of the fruit in pepper. Understanding how these genes are differentially regulated in both species might be a key issue for the agronomic improvement of prickly eggplant varieties.

In addition to contributing to their visual attraction, the accumulation of anthocyanins in fruits is an important nutritional factor for the human diet and is a trait widely studied in members of the Solanaceae family (Spelt et al., 2000; Doganlar et al., 2002a; Mathews et al., 2003; Borovsky et al., 2004; De Jong et al., 2004; Gonzali et al., 2009; Barchi et al., 2012; Portis et al., 2014). A major QTL located in a conserved region on lower chromosome 10 was detected in several studies of Solanaceae family members. This was confirmed in eggplant by Doganlar et al. (2002a), Barchi et al. (2012), and Portis et al. (2014), who identified several major QTL at the same locus related to anthocyanin pigmentation. The three studies suggest the presence of a single pleiotropic gene influencing several traits, rather than multiple independent loci. Other analyses for candidate genes carried out in several Solanaceae isolated a highly conserved transcription factor of the MYB family which was called, respectively, anthocyanin2 (AN2) in petunia (Borovsky et al., 2004), anthocyanin1 (ANT1 or AN1) in tomato (Mathews et al., 2003) and just anthocyanin (A gene) in pepper (Chaim et al., 2003; Borovsky et al., 2004). Interestingly, by aligning the sequences of AN2, ANT1, and A gene with the pepper genome, using Blastn, we confirmed a locus on lower P10 (~183 Mbp) at which each of these genes co-localized. In the same region (~182.5 Mbp) two aligned eggplant markers (15158_PstI_L379 and 19126_PstI_L349, see Supplementary Table S3, Figures 5A,B) related with QTL strongly influencing the accumulation of anthocyanin in the leaf, peduncle, stem, and calix. This provides support for the presence in Solanaceae species of an orthologous region controlling the trait (De Jong et al., 2004). Furthermore, three other loci related on anthocyanin content were found on upper P11 and lower P06 and P12 (Supplementary Table S3).

## Conclusions

Our results demonstrate that the increasing availability of genomic tools and of physical maps for crop plants permits highly detailed syntenic analyses among related plant species.

This is the case for pepper and tomato, where we were able to perform an in-depth analysis of synteny and to identify previously reported as well as newly detected chromosomal rearrangements which occurred during lineage into their current forms.

However, due to the availability of only a rather fragmented eggplant genome sequence, of which only about 12% was anchored, we were not able to identify the physical position of QTL we had previously located in an intraspecific eggplant genetic map. This led us to conduct syntenic analysis between eggplant and both pepper and tomato by aligning a high resolution genetic map of the former with the publicly available genome sequence of the latter. The results we obtained demonstrate the lower resolution of comparative genome studies achievable with this approach. Notwithstanding, following the alignment of an eggplant marker dataset with both the tomato and pepper genome sequences we confirmed most of the rearrangements previously identified, and were able to detect putative new ones.

A further step of our study was to identify QTL orthology between eggplant and pepper. This was achieved by locating the position on the pepper genome of eggplant (Barchi et al., 2012; Portis et al., 2014, 2015) and pepper markers (Yarnes et al., 2013) associated with QTL influencing key breeding traits, while eggplant QTL lying on translocated chromosomal portions were validated by the syntenic analysis we had previously performed. Overall, we found 152 eggplant QTL orthologous to 151 pepper QTL, and to our knowledge, these results represent the first direct assessment of orthology between the two species.

As future perspectives, candidate gene analyses will be performed to identify the gene sequences lying on QTL confidence intervals. The future availability of an high quality eggplant genome sequence will improve the resolution of syntenic analyses of eggplant with tomato and pepper.

## Author contributions

AV, HA, SL planned and supervised the experimental work; TH, HA, and AV developed the genetic map of pepper and identified the QTL used in this study; LB, EP, LT, and GLR developed the genetic map of eggplant and identified the QTL used in this study; RR and HA performed sequence alignments and development of Pepper Gbrowse; RR and TH performed the synteny establishment between tomato, pepper and eggplant; RR performed programming and evaluated the associations between pepper and eggplant QTL; SL, RR, EP, TH, and LB drafted the manuscript; all authors read and approved the final version of the manuscript.

### Conflict of interest statement

The authors declare that the research was conducted in the absence of any commercial or financial relationships that could be construed as a potential conflict of interest.

## Supplementary material

Supplementary Table S1 **List of eggplant QTL identified by Barchi et al. (2012) and Portis et al. (2014, 2015)**.

Supplementary Table S2 **The pepper QTL identified by Yarnes et al. (2013)**.

Supplementary Table S3 **The eggplant markers aligned on pepper genome sequence**.

Supplementary Table S4 **The pepper markers aligned on pepper genome sequence**.

Supplementary Table S5 **The eggplant and pepper markers grouped by clusters**.

Supplementary Table S6 **The table shows the clusters observed in the alignment of eggplant and pepper markers**. The eggplant traits are given in the left column while on the right the pepper traits included in each cluster are reported.

Supplementary Figure S1 **Details of chromosome synteny between tomato CDS and pepper genome**. Physical positions of tomato CDS matching pepper genome in detail. The relative position of tomato CDS is shown on the ordinate axis while on the abscissa axis there are the normalized pepper chromosomes. The captions on the right show the chromosome of origin of tomato CDS. Physical positions of COSII markers matching tomato and pepper genome sequence are shown by the red and yellow stars. The inversions previously reported in literature are marked with an orange asterisk, while the newly identified inversions are marked with a red asterisk.

Supplementary Figure S2 **QTL location (only loci harboring QTL are shown)**. The figure is complementary with the Supplementary Table S5 and shows the loci bearing both eggplant and pepper markers. The scale shown on the Y axis indicates the LOD score. The scale on the X axis indicates the position on pepper chromosome in Mbps. Map positions of the QTL are given by the peaks formed by each marker and the confidence interval is indicated by the width of the lines. Black dots indicate the position of eggplant markers associated to QTL. Pepper markers are reported to the right of the figures.
