# Supplementary material for: New Insights on Eggplant/Tomato/Pepper Synteny and Identification of Eggplant and Pepper Orthologous QTL
Source: Front Plant Sci. 2016 Jul 18;7:1031. doi: 10.3389/fpls.2016.01031 (PMC4948011; doi:10.3389/fpls.2016.01031)
Supplement: Supplementary Figure S1 — Details of chromosome synteny between tomato CDS and pepper genome. Physical positions of tomato CDS matching pepper genome in detail. The relative position of tomato CDS is shown on the ordinate axis while on the abscissa axis there are the normalized pepper chromosomes. The captions on the right show the chromosome of origin of tomato CDS. Physical positions of COSII markers matching tomato and pepper genome sequence are shown by the red and yellow stars. The inversions previously reported in literature are marked with an orange asterisk, while the newly identified inversions are marked with a red asterisk. [file Image1.PDF]

Supplemental Figure 1

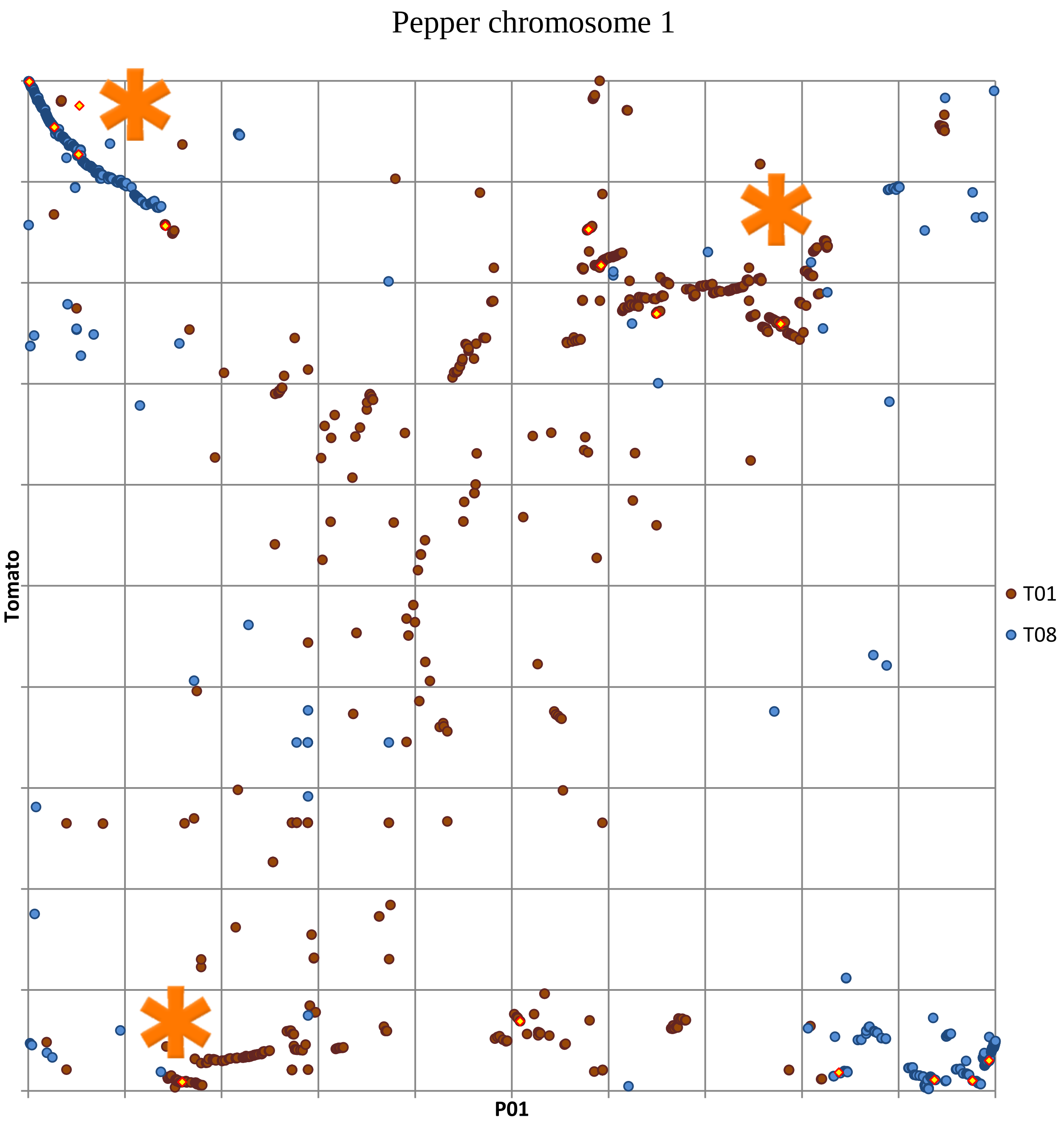

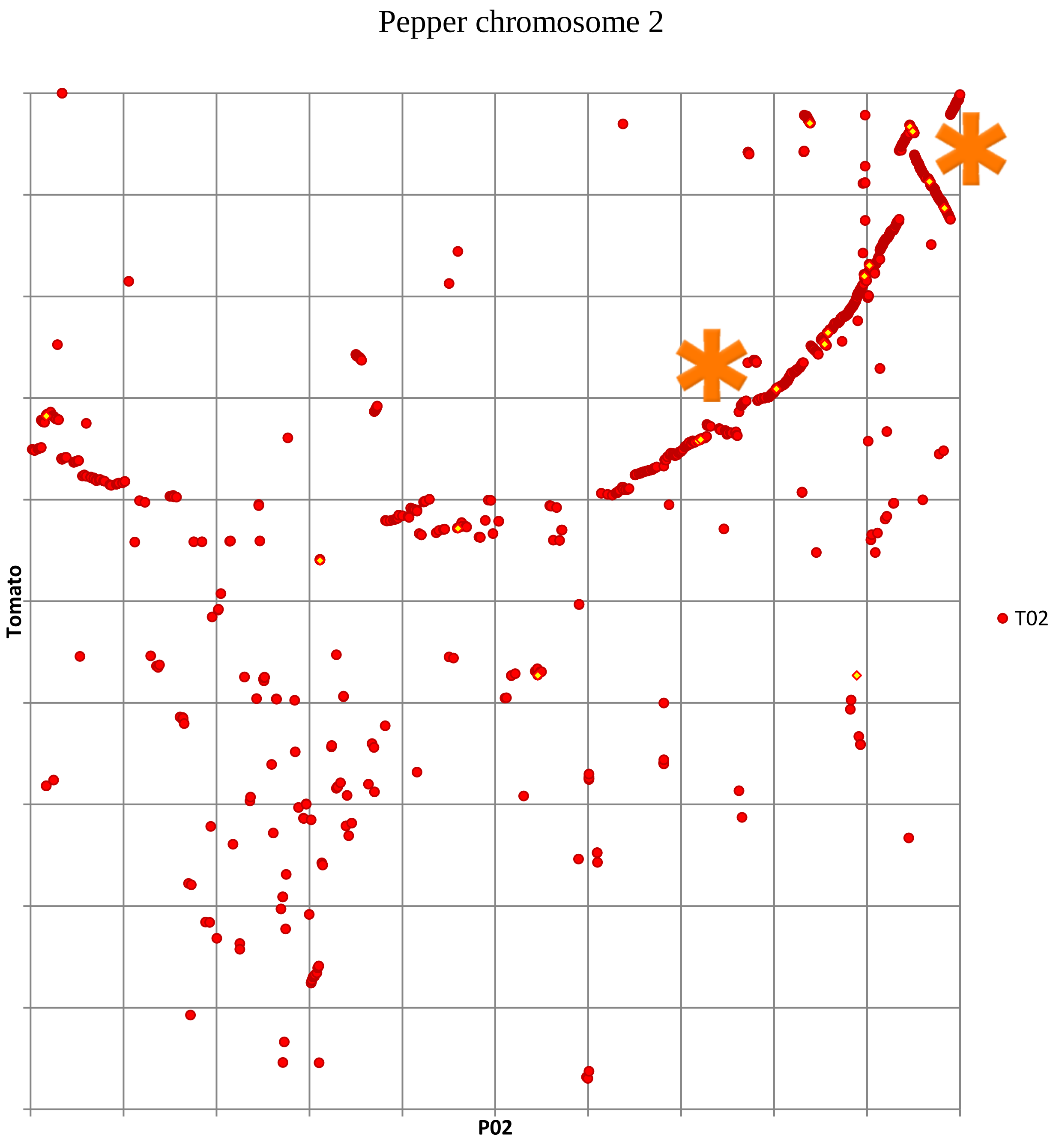

Pepper chromosome 2
Tomato
P02
T02

Pepper chromosome 3

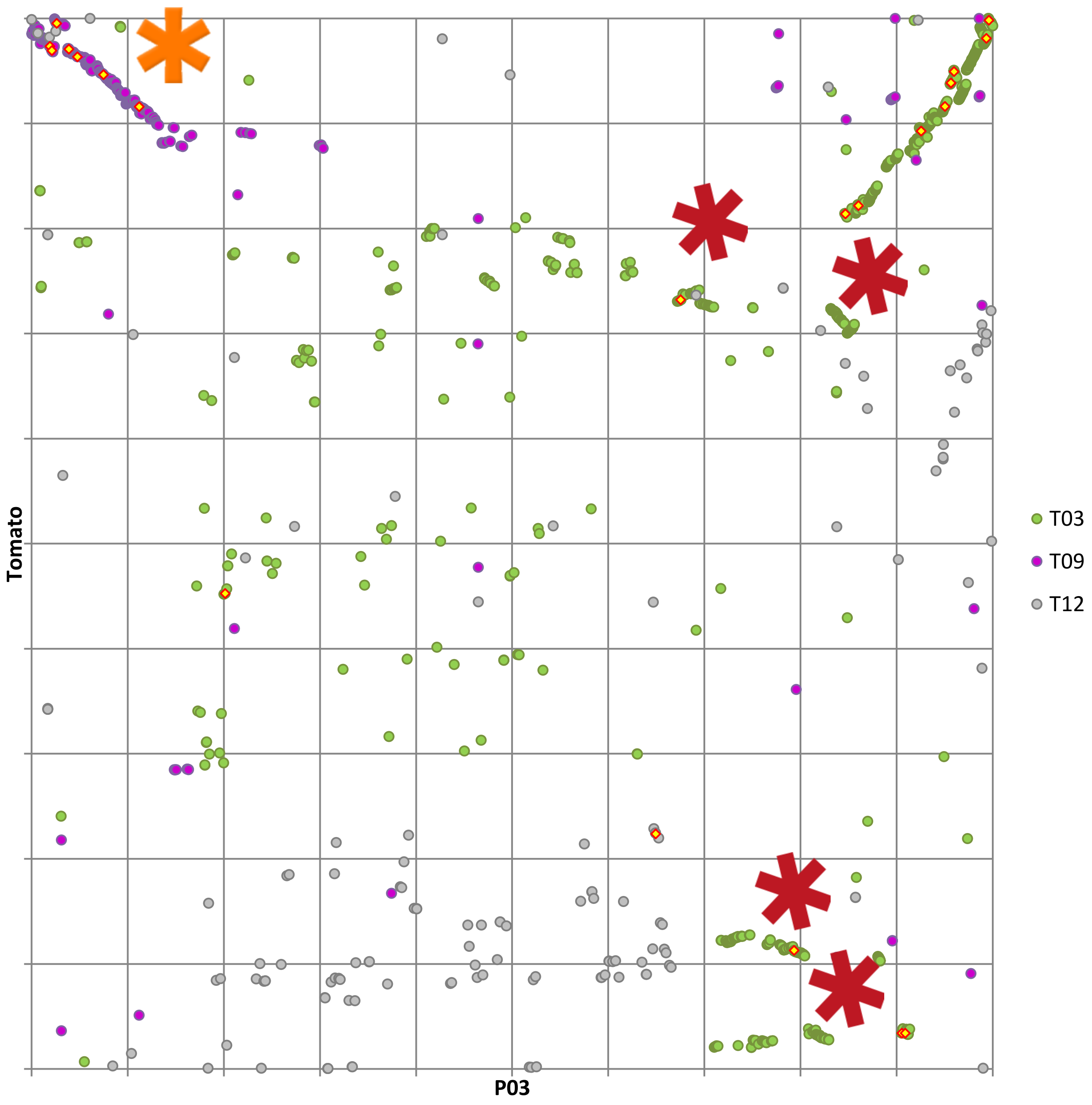

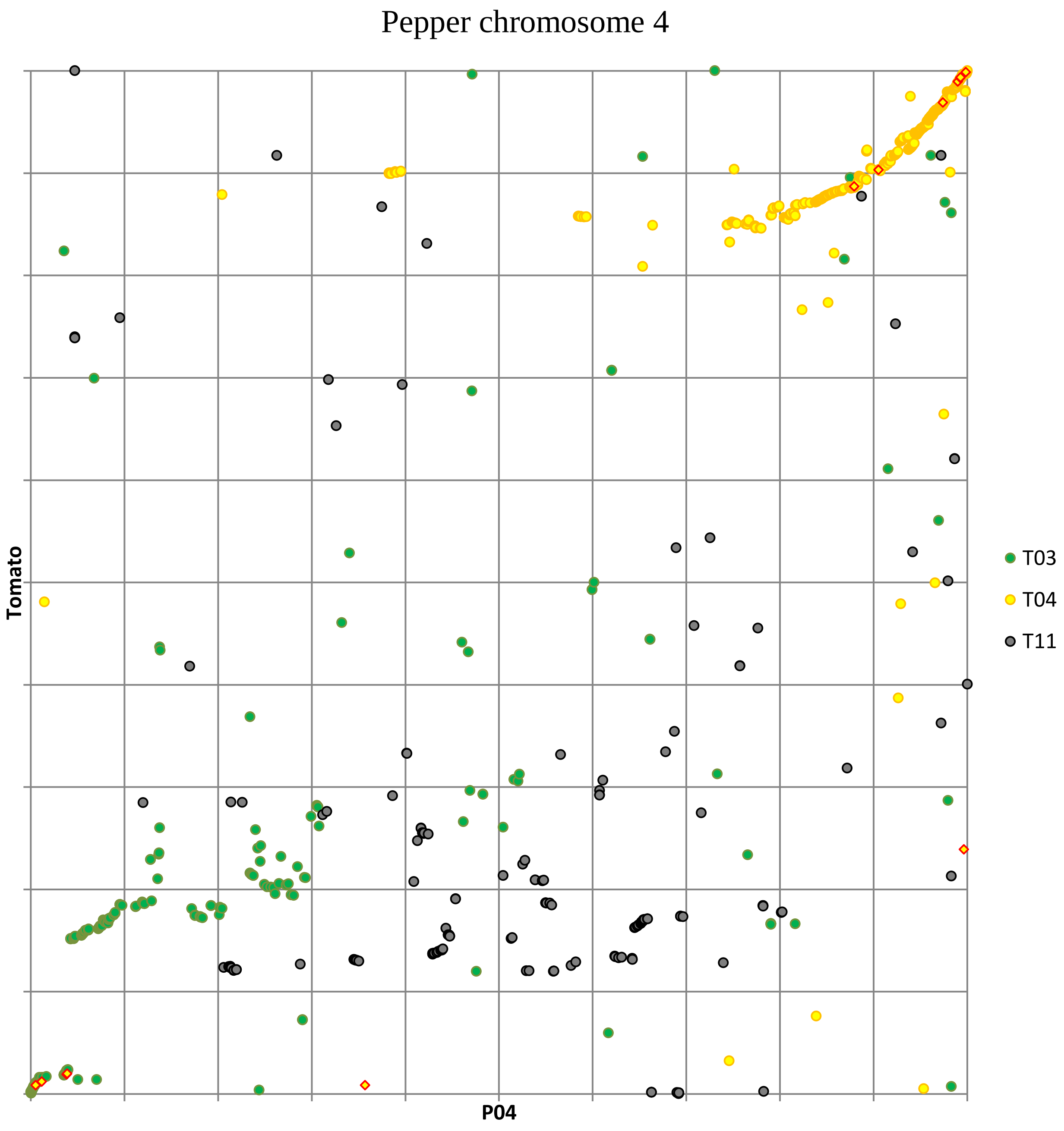
Pepper chromosome 4
Tomato
P04
T03
T04
T11

Pepper chromosome 5

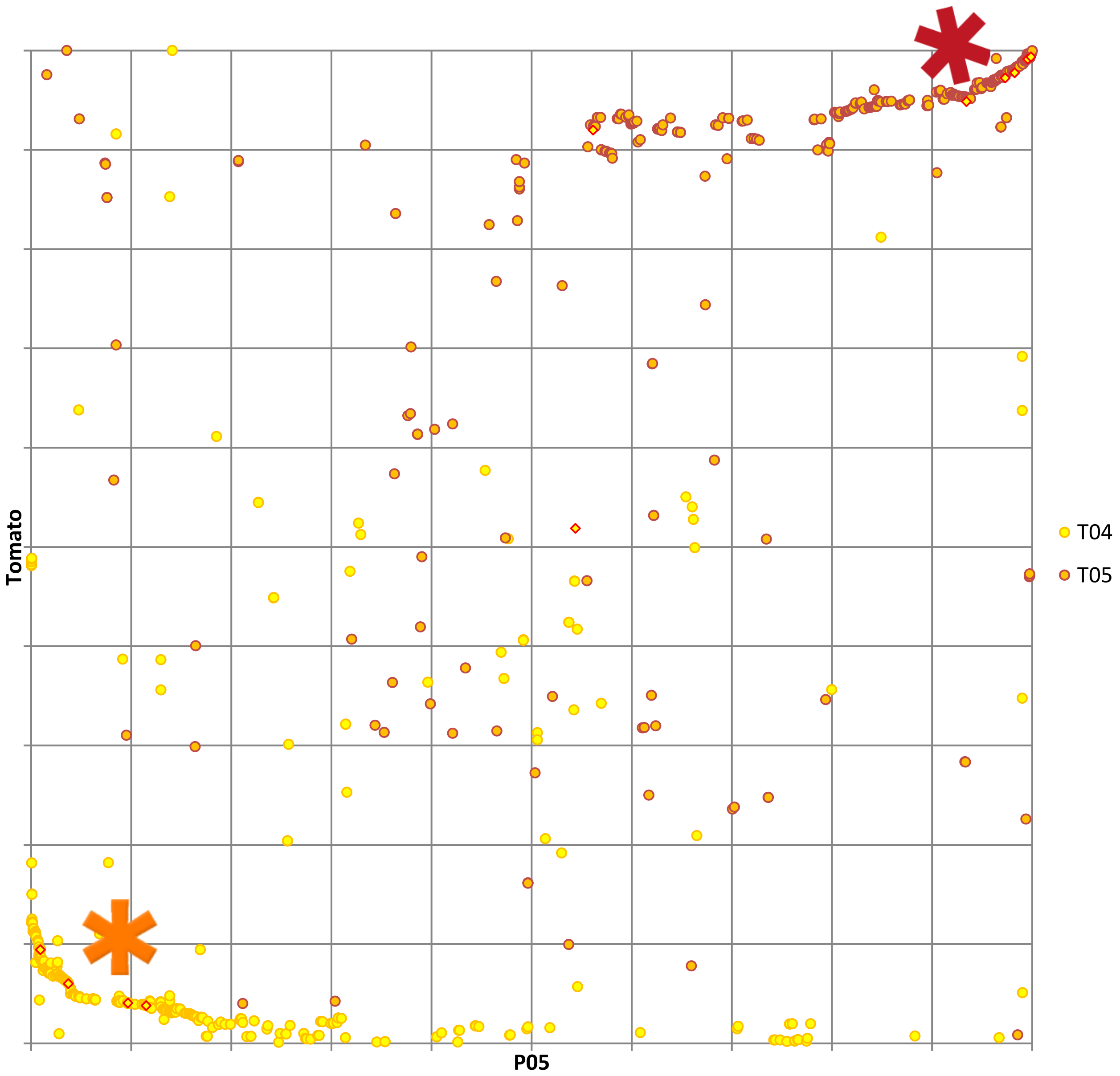

Pepper chromosome 6

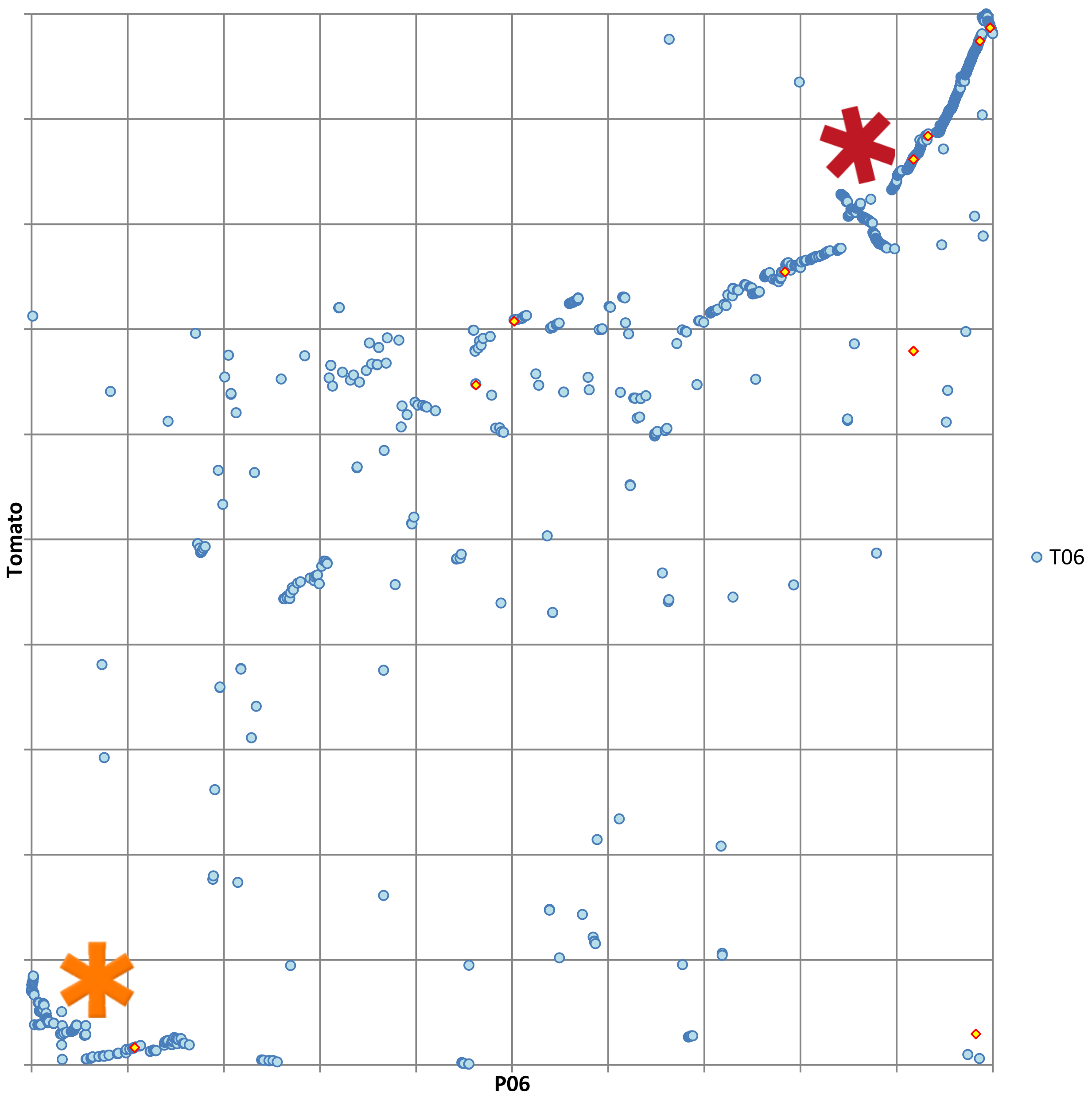

Pepper chromosome 7

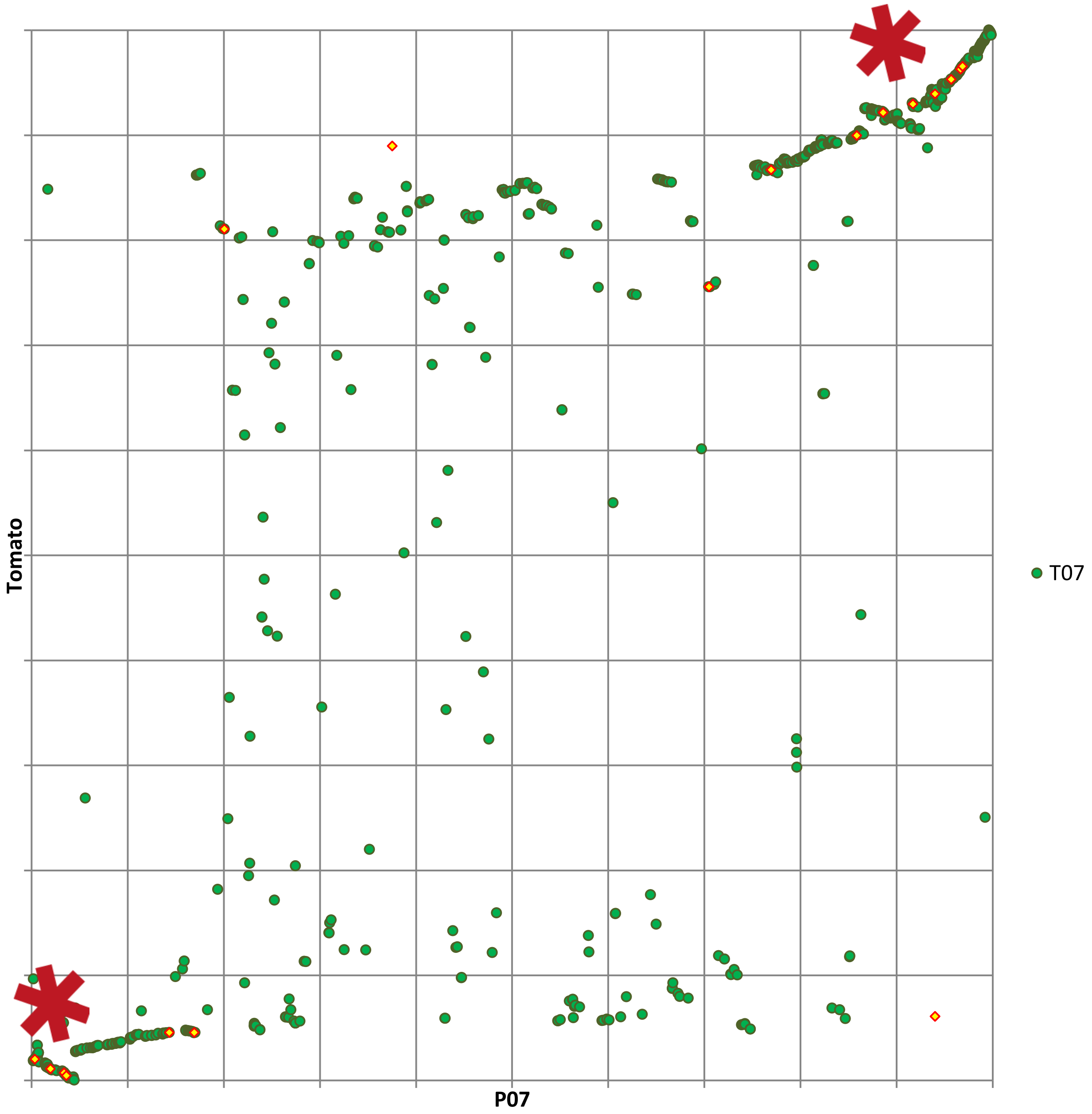

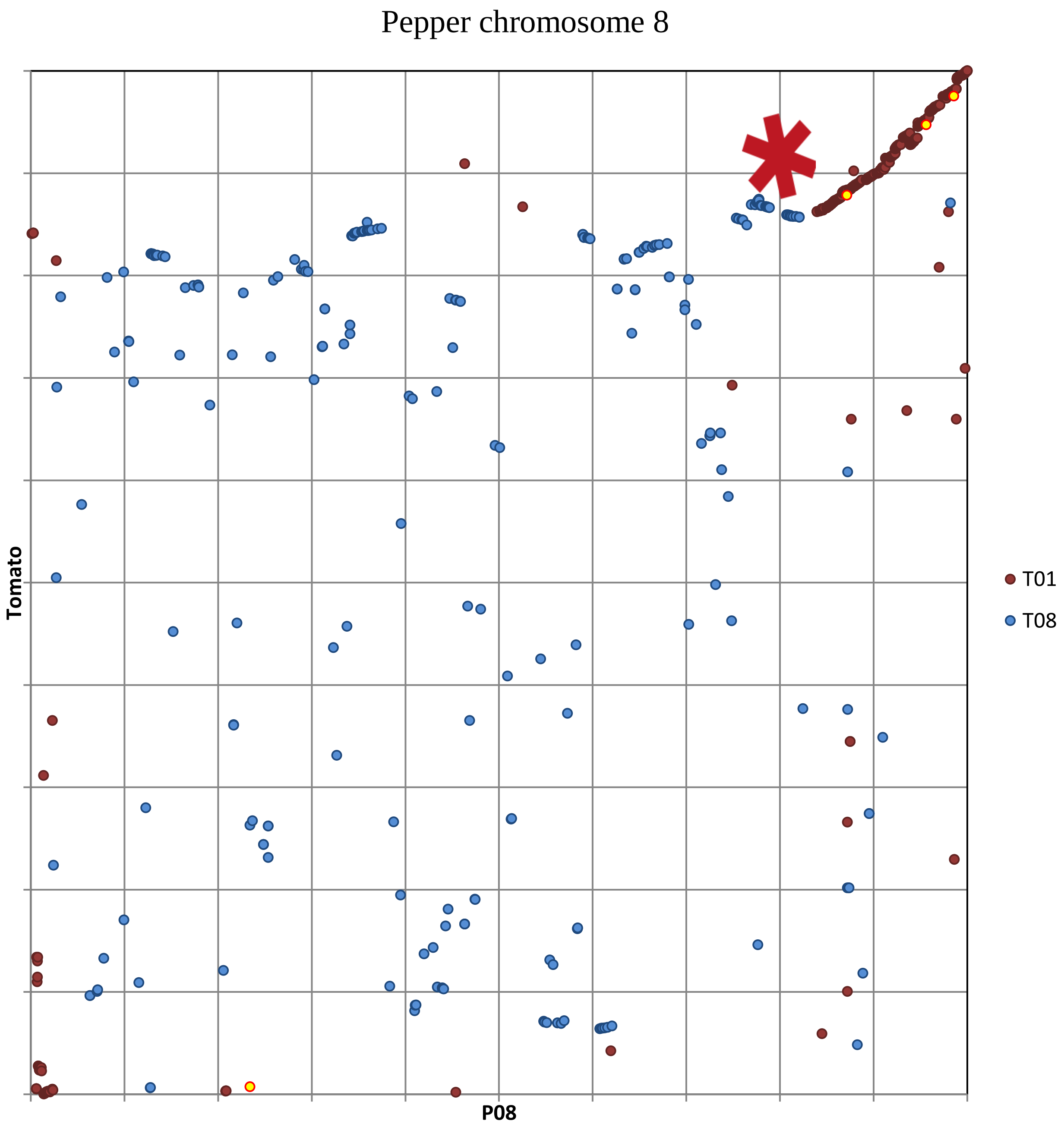

Pepper chromosome 8
Tomato
P08
T01
T08

Pepper chromosome 9

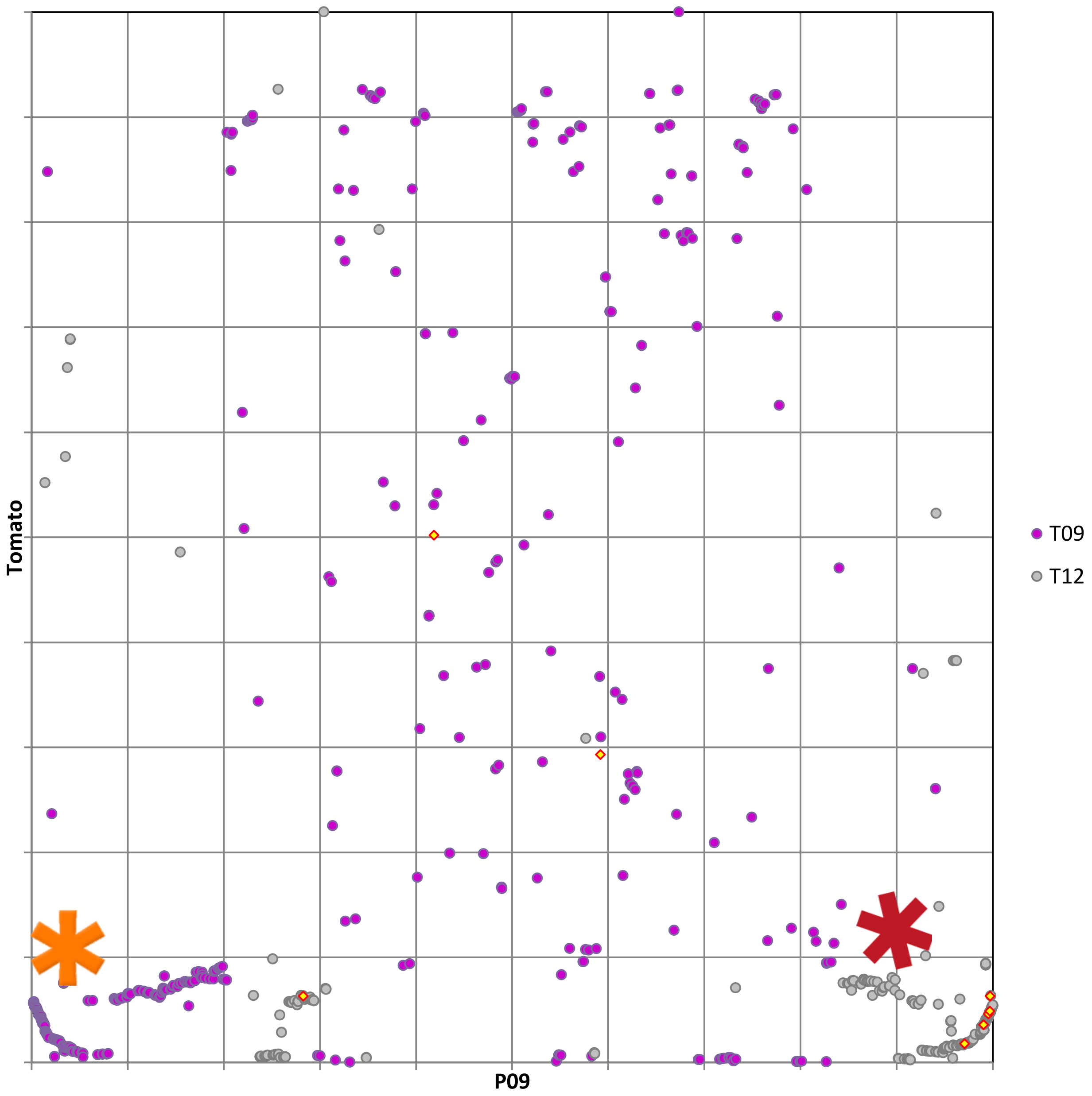

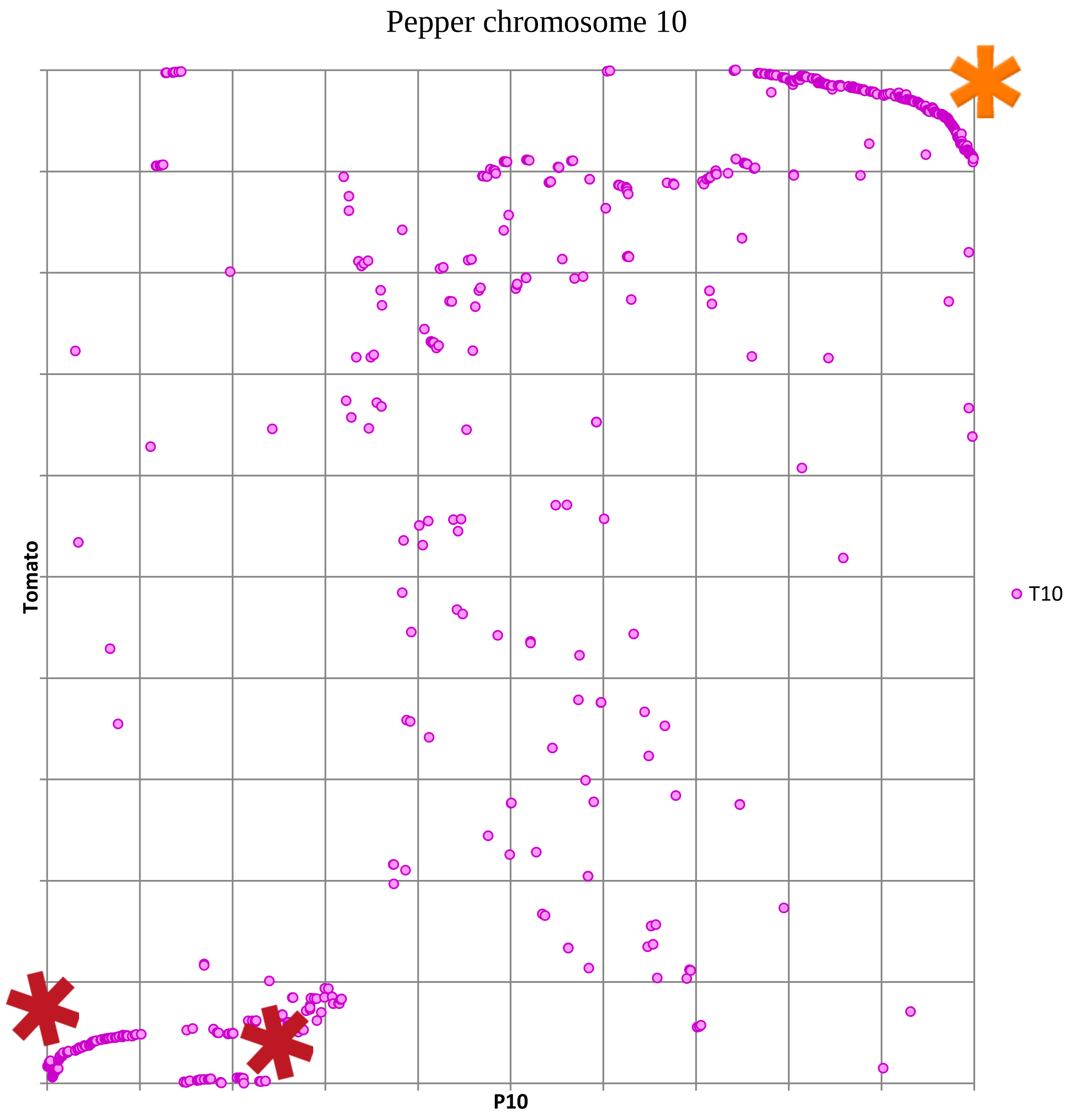

Pepper chromosome 10
Tomato
P10
T10

Pepper chromosome 11

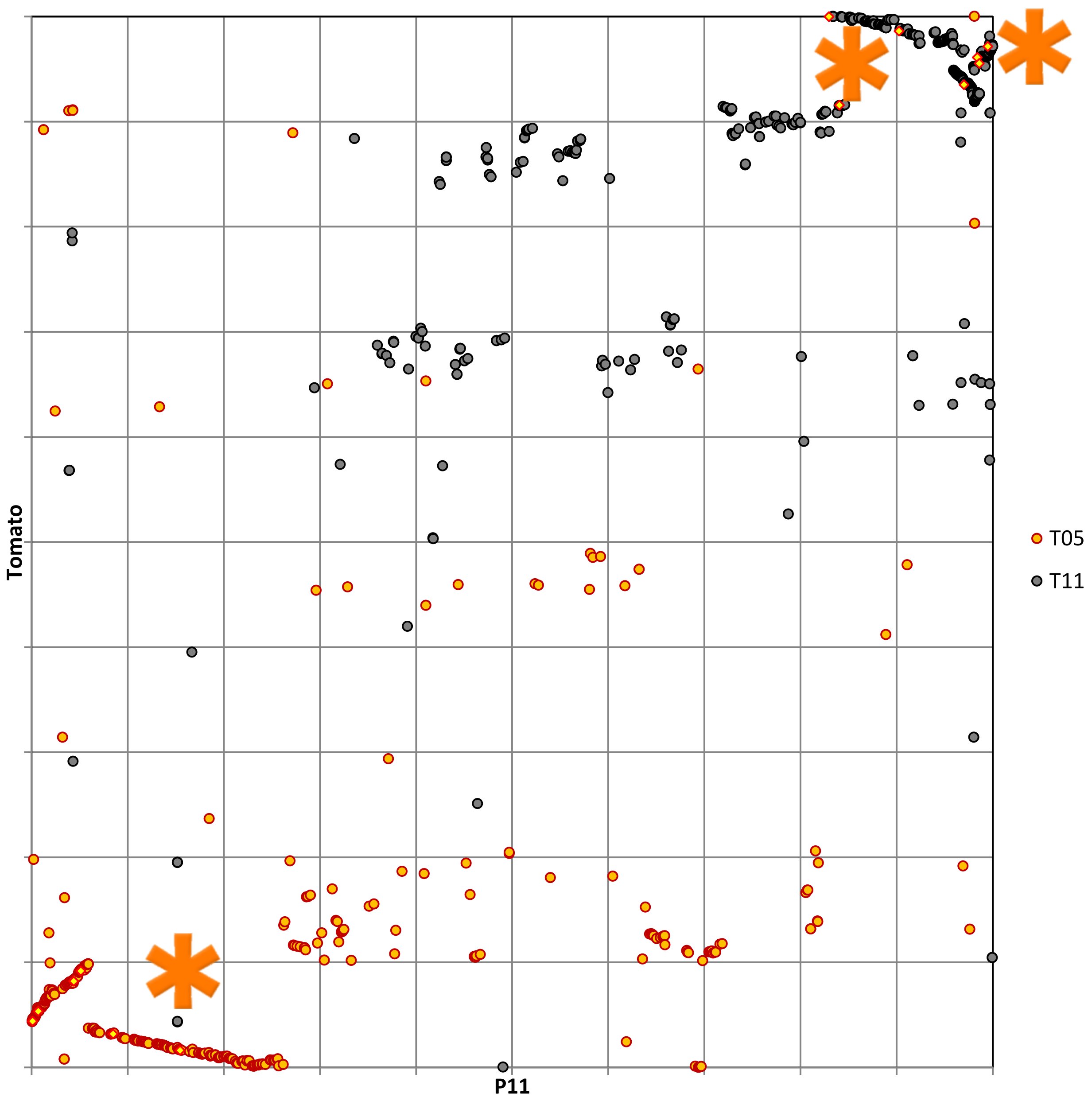

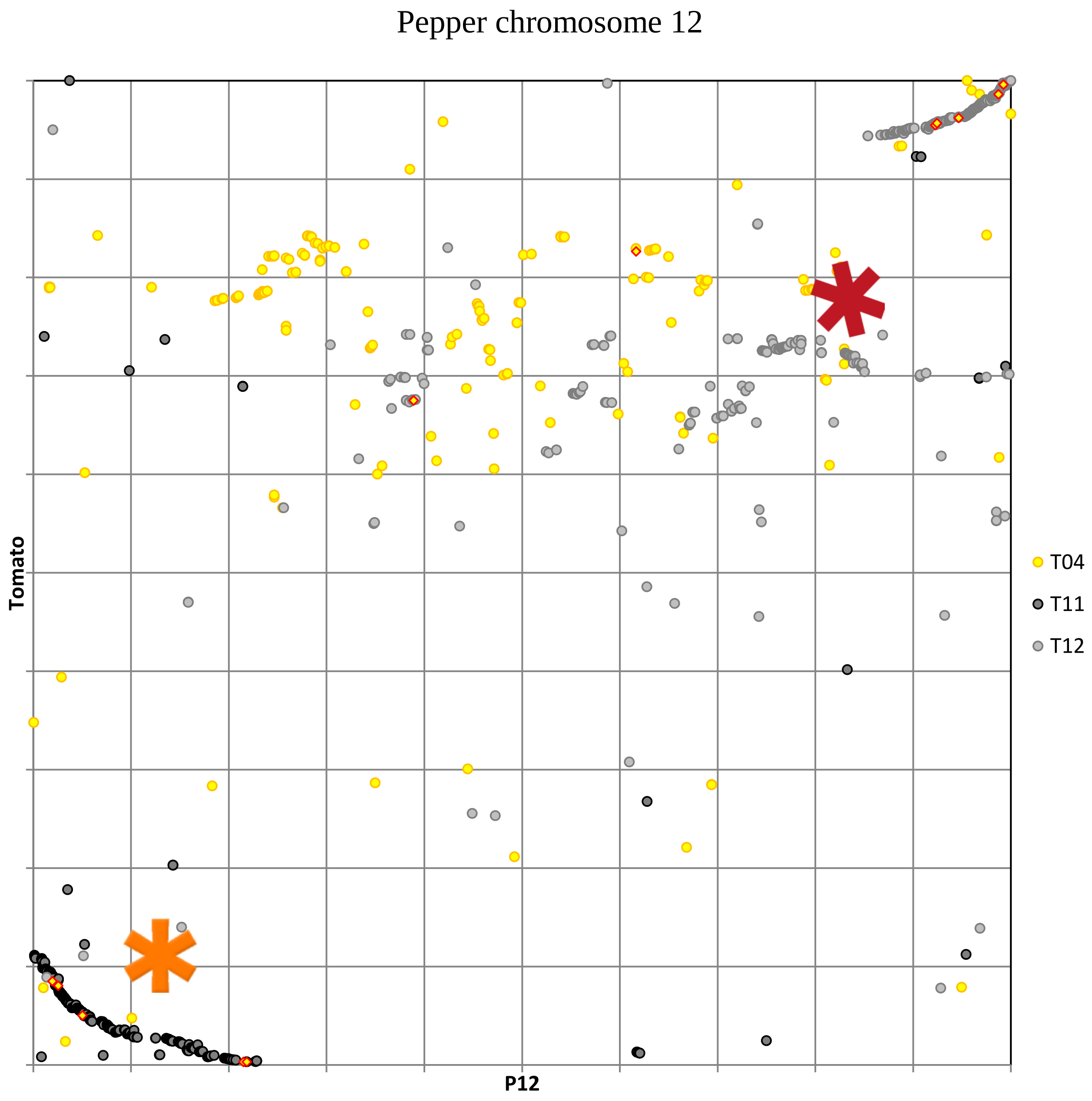

Figure S2. Details of chromosome synteny between tomato CDS and pepper genome. Physical positions of tomato CDS matching pepper genome in detail. The relative position of tomato CDS is shown on the ordinate axis while on the abscissa axis there are the normalized pepper chromosomes. The captions on the right show the chromosome of origin of tomato CDS. Physical positions of COSII markers matching tomato and pepper genome sequence are shown by the red and yellow stars.
