# Supplementary figures and images for: New Insights on Eggplant/Tomato/Pepper Synteny and Identification of Eggplant and Pepper Orthologous QTL
Source: Front Plant Sci. 2016 Jul 18;7:1031. doi: 10.3389/fpls.2016.01031 (PMC4948011; doi:10.3389/fpls.2016.01031)

Supplemental Figure 2

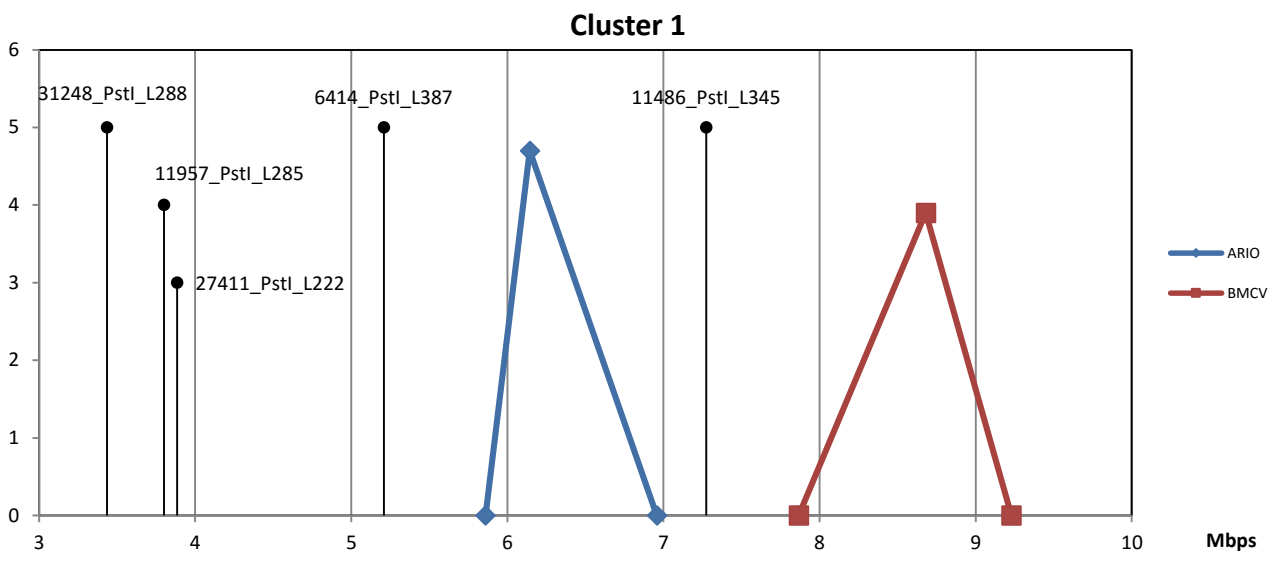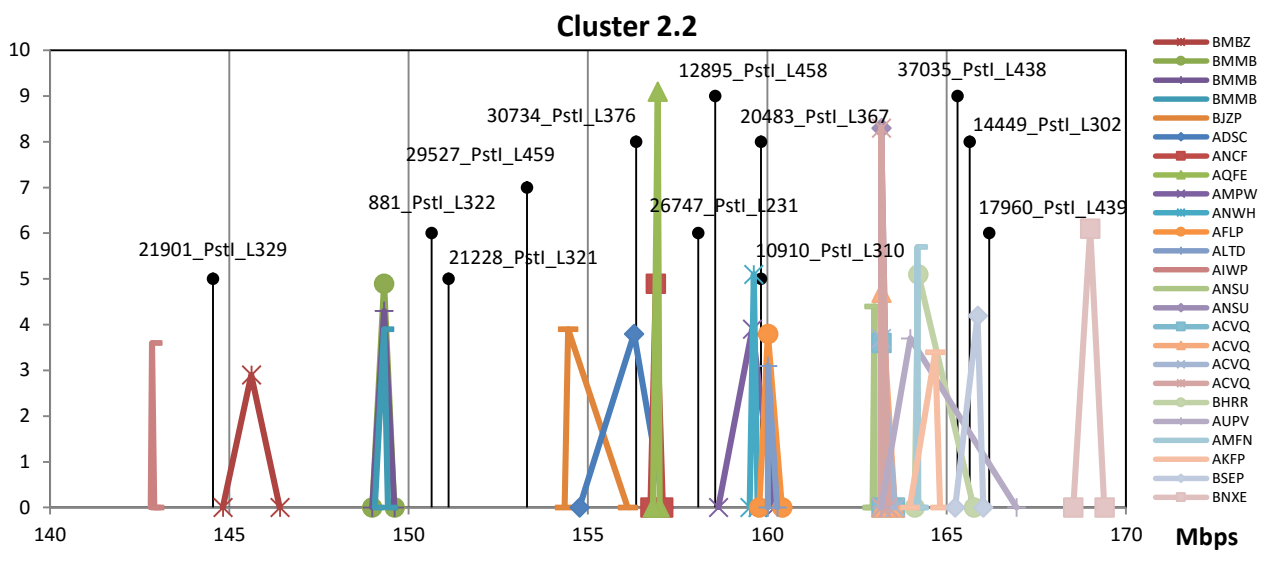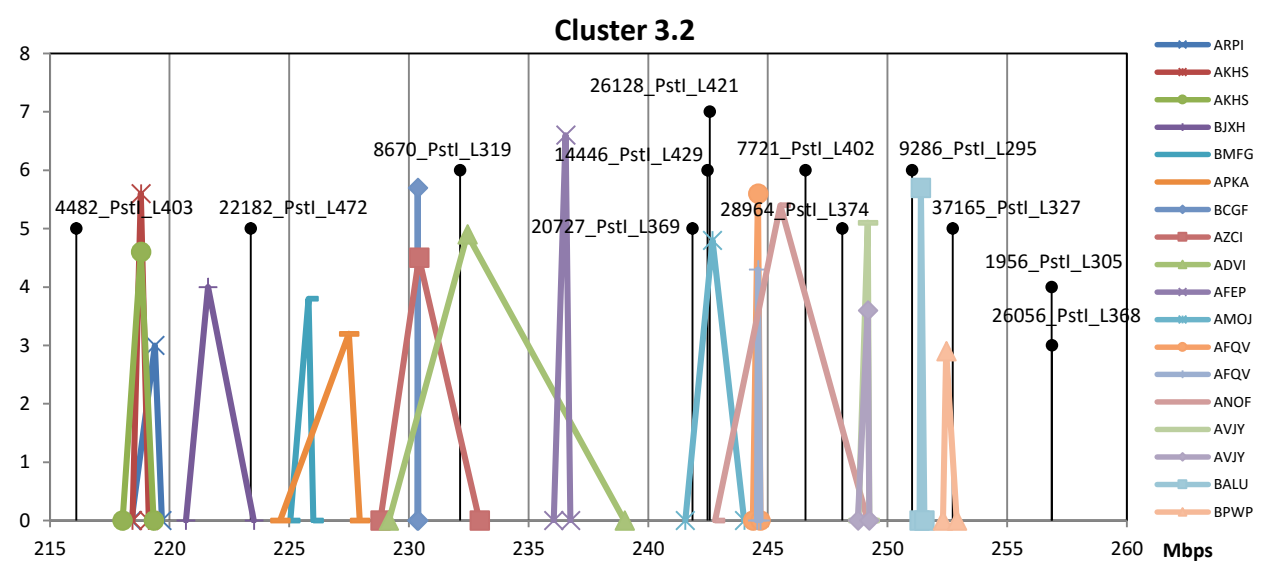

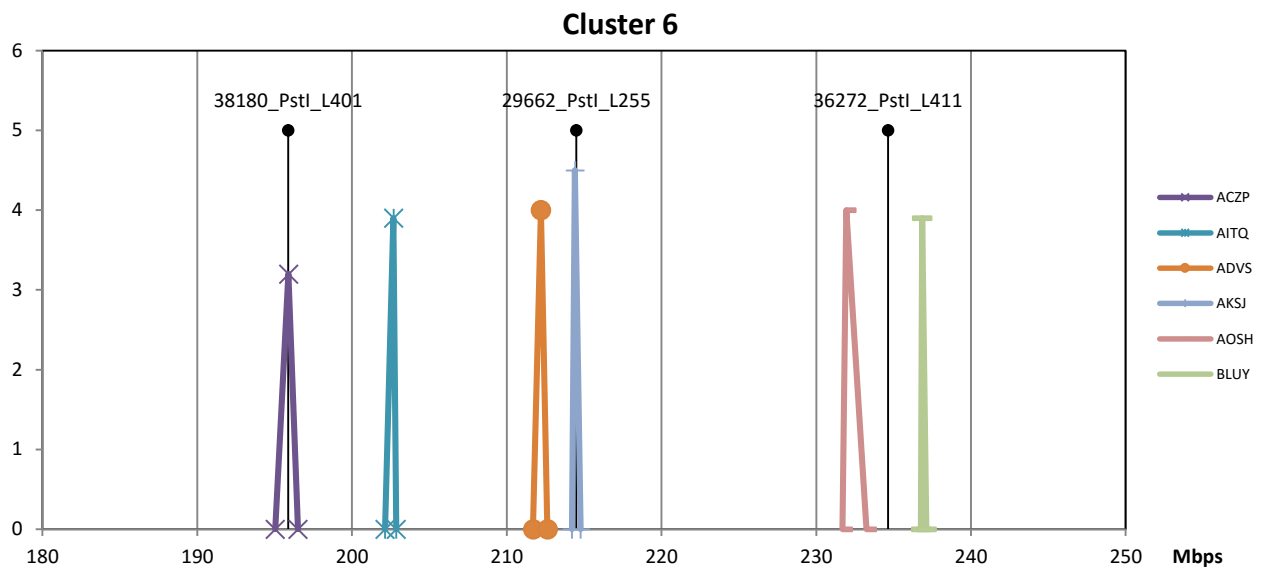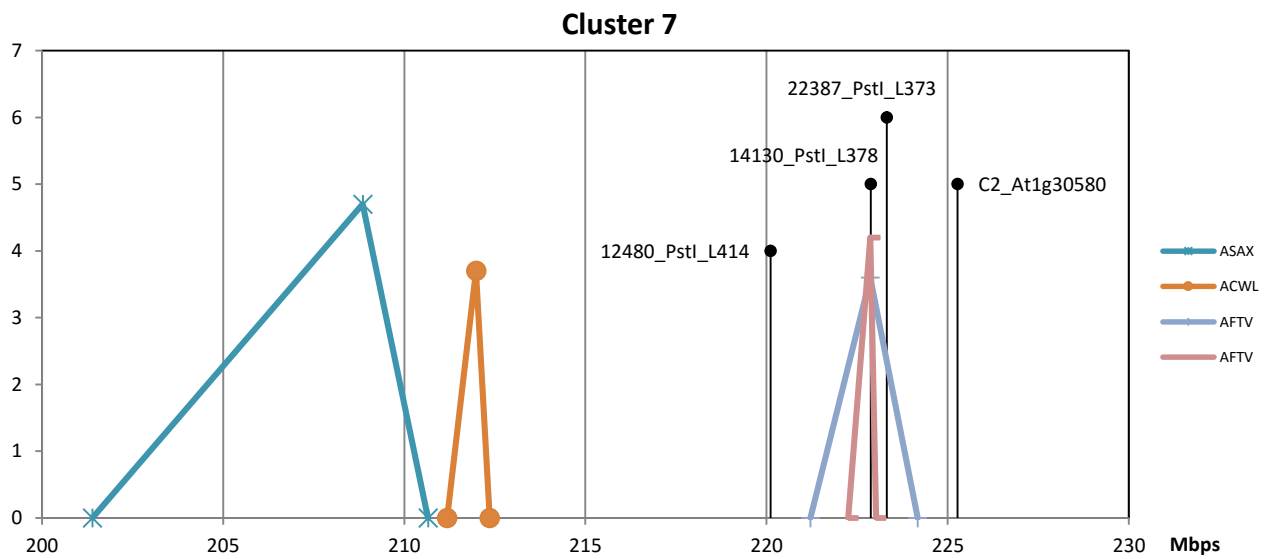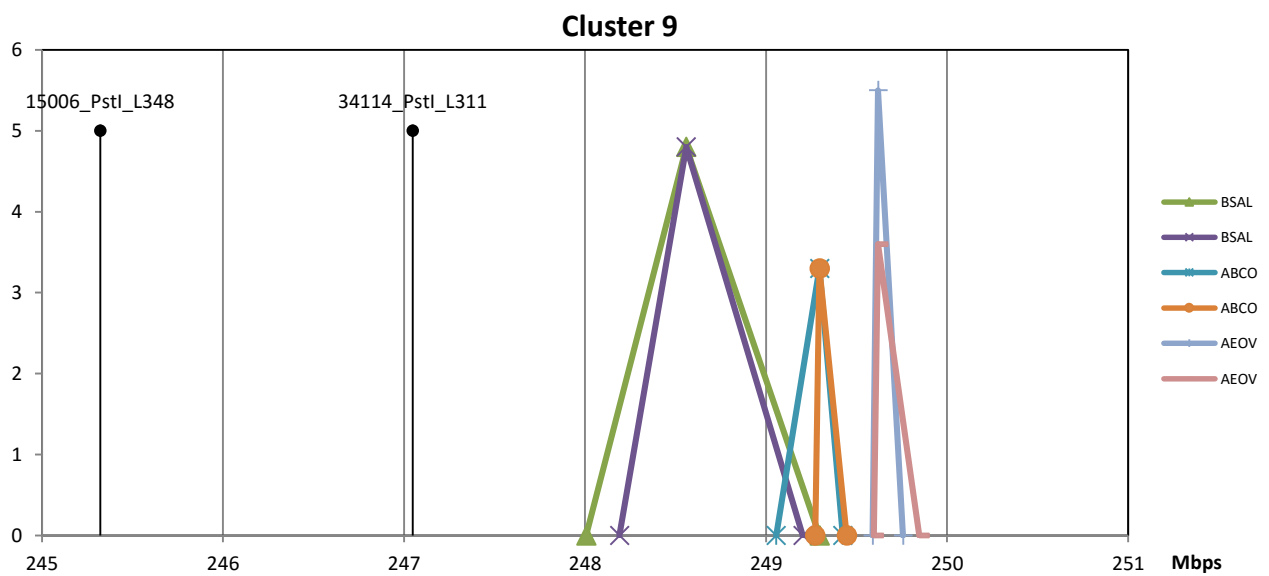

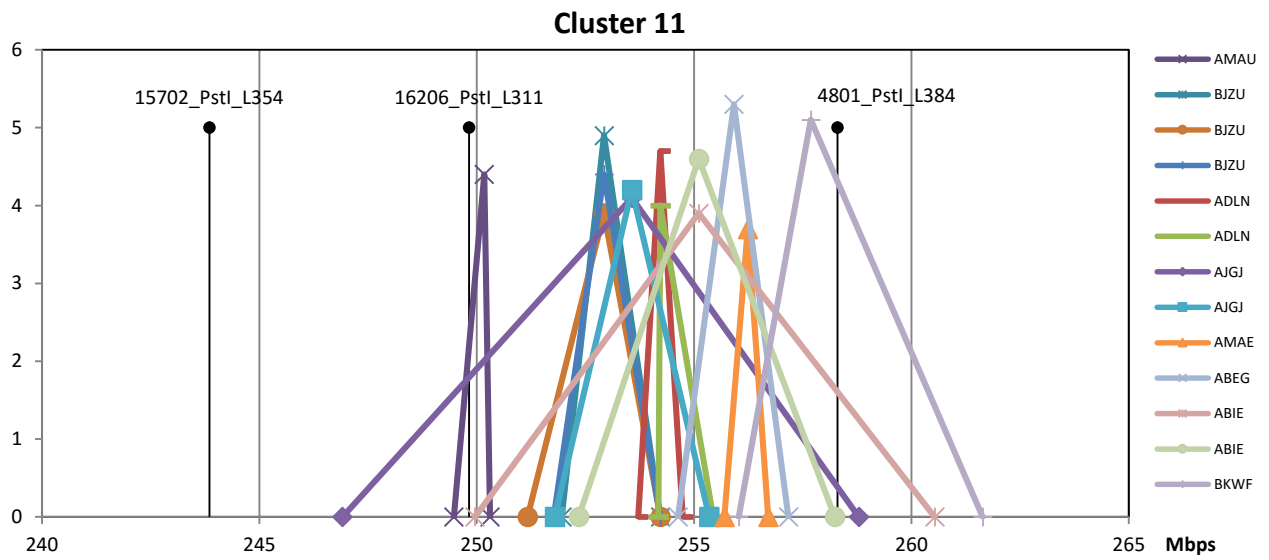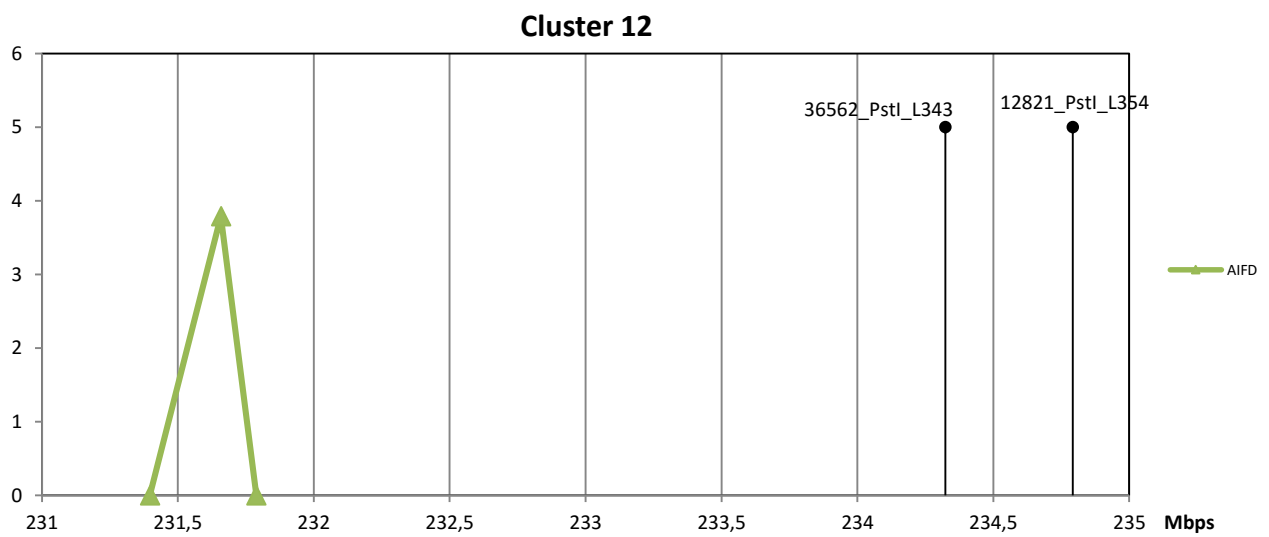

Supplement: Supplementary Figure S2 — QTL location (only loci harboring QTL are shown). The figure is complementary with the Supplementary Table S5 and shows the loci bearing both eggplant and pepper markers. The scale shown on the Y axis indicates the LOD score. The scale on the X axis indicates the position on pepper chromosome in Mbps. Map positions of the QTL are given by the peaks formed by each marker and the confidence interval is indicated by the width of the lines. Black dots indicate the position of eggplant markers associated to QTL. Pepper markers are reported to the right of the figures. [file Image2.PDF]
